# Development and comparative validation of genomic-driven PCR-based assays to detect *Xanthomonas citri* pv. citri in citrus plants

**DOI:** 10.1186/s12866-020-01972-8

**Published:** 2020-10-01

**Authors:** Isabelle Robène, Véronique Maillot-Lebon, Aude Chabirand, Aurélie Moreau, Nathalie Becker, Amal Moumène, Adrien Rieux, Paola Campos, Lionel Gagnevin, Myriam Gaudeul, Claudia Baider, Fréderic Chiroleu, Olivier Pruvost

**Affiliations:** 1 CIRAD, UMR PVBMT, Saint-Pierre, Reunion Island France; 2 Unit for Tropical Pests and Diseases, Plant Health Laboratory (LSV), French Agency for Food, Environmental and Occupational Health & Safety (ANSES), Saint-Pierre, Reunion Island France; 3 Institut de Systématique, Evolution, Biodiversité (ISYEB), Muséum National d’Histoire Naturelle, Sorbonne Université, EPHE, Université des Antilles, CNRS, Paris, France; 4 Université de La Réunion, UMR PVBMT, Saint-Pierre, Reunion Island France; 5 CIRAD-UMR IPME, Montpellier, France; 6 Herbier national (P), Muséum National d’Histoire Naturelle, Paris, France; 7 Ministry of Agro Industry and Food Security, Mauritius Herbarium, R.E. Vaughan Building (MSIRI compound) Agricultural Services, Réduit, Mauritius

**Keywords:** Asiatic Citrus canker, Surveillance, Real-time quantitative PCR, Diagnostics, Cycle cut-off, Ancient DNA

## Abstract

**Background:**

Asiatic Citrus Canker, caused by *Xanthomonas citri* pv. citri, severely impacts citrus production worldwide and hampers international trade. Considerable regulatory procedures have been implemented to prevent the introduction and establishment of *X. citri* pv. citri into areas where it is not present. The effectiveness of this surveillance largely relies on the availability of specific and sensitive detection protocols. Although several PCR- or real-time PCR-based methods are available, most of them showed analytical specificity issues. Therefore, we developed new conventional and real-time quantitative PCR assays, which target a region identified by comparative genomic analyses, and compared them to existing protocols.

**Results:**

Our assays target the *X. citri* pv. citri XAC1051 gene that encodes for a putative transmembrane protein. The real-time PCR assay includes an internal plant control (5.8S rDNA) for validating the assay in the absence of target amplification. A receiver-operating characteristic approach was used in order to determine a reliable cycle cut-off for providing accurate qualitative results. Repeatability, reproducibility and transferability between real-time devices were demonstrated for this duplex qPCR assay (XAC1051-2qPCR). When challenged with an extensive collection of target and non-target strains, both assays displayed a high analytical sensitivity and specificity performance: LOD_95%_ = 754 CFU ml^− 1^ (15 cells per reaction), 100% inclusivity, 97.2% exclusivity for XAC1051-2qPCR; LOD_95%_ = 5234 CFU ml^− 1^ (105 cells per reaction), 100% exclusivity and inclusivity for the conventional PCR. Both assays can detect the target from naturally infected citrus fruit. Interestingly, XAC1051-2qPCR detected *X. citri* pv. citri from herbarium citrus samples. The new PCR-based assays displayed enhanced analytical sensitivity and specificity when compared with previously published PCR and real-time qPCR assays.

**Conclusions:**

We developed new valuable detection assays useful for routine diagnostics and surveillance of *X. citri* pv. citri in citrus material. Their reliability was evidenced through numerous trials on a wide range of bacterial strains and plant samples. Successful detection of the pathogen was achieved from both artificially and naturally infected plants, as well as from citrus herbarium samples, suggesting that these assays will have positive impact both for future applied and academic research on this bacterium.

## Background

Over the last half century, there has been a dramatic increase in biological invasions worldwide as a result of globalization and increased international trade and travel [1]. To limit the threat posed by the introduction of exotic plant pathogens and pests through trade and human transport, many countries have tightened border biosecurity surveillance, as well as phytosanitary inspection, and quarantine measures. This biosecurity effort has slowed down the introduction and establishment of pathogens despite the increase in trade and the international movement of people. However, biosecurity measures have adopted to differing degrees across the agricultural sector. Measures to protect annual crop and pasture species have had a positive impact. In contrast, the biosecurity effort should be enhanced for perennial crops such as forest and fruit tree species, which remain vulnerable [2]. When a plant disease outbreak is observed in a new area, quick and appropriate management measures, such as containment or eradication, are necessary to avoid the establishment and further spread of the pathogen. For efficient biosecurity surveillance and plant disease management, identifying regulated pathogens fast and accurately is essential. Misdiagnosis can have a severe economic impact, for example, if unwanted pathogens are introduced or inappropriate management options applied [3–5].

*Xanthomonas citri* pv. citri, the causal agent of Asiatic citrus canker (ACC), is an example of a high-concern regulated pathogen that threatens an economically important fruit crop (i.e., collectively, citrus fruit rank #1 fruit crop worldwide). This bacterial pathogen causes serious direct and indirect economic losses due to reduced crop yield and quality, the cost of eradication, containment (including the destruction of nursery, grove or backyard trees) or integrated management measures, as well as embargos on the movement of fruit. In addition, it raises concerns about environmental issues linked to increased pesticide use and the development of resistance to antimicrobials [4, 6]. For example, more than one billion US dollars was spent over a decade in Florida in an attempt to eradicate the pathogen [7]. Australia organized several successful ACC eradication campaigns in the past [8, 9], and is currently conducting a response plan in the Northern Territory at a cost of millions of A$.

Although at least four distinct xanthomonads are pathogenic to citrus, only two of them, *X. citri* pv. citri and *X. citri* pv. aurantifolii, cause visually indistinguishable canker-like symptoms. They are the causal agents of Asiatic and South American citrus canker, respectively. However, only the former bacterium has a major agricultural significance, because it is the only one associated with serious canker outbreaks even in countries where both pathovars occur concomitantly. Within *X. citri* pv. citri, strains differ in host range among citrus lines and can be classified into three distinct pathotypes. Pathotype A strains have the greatest global economic impact on the citrus industry. They are widely distributed and induce canker on a broad range of rutaceous hosts, including many *Citrus* species, hybrids or related genera such as trifoliate orange (*Poncirus trifoliata*) [4]. Pathotype A* strains are pathogenic to a restricted range of citrus hosts. Most outbreaks occur on Mexican lime (*Citrus* x *aurantiifolia*) in Asia, the Arabian Peninsula and Eastern Africa [10–12]. Pathotype A^w^ has been reported to date on the Indian subcontinent, in the Arabian Peninsula and the USA. Natural infections are restricted to Mexican lime and alemow (*C.* x *macrophylla*). It has the unique feature of causing a hypersensitive response when inoculated into some non-host citrus lines such as *C.* x *paradisi* and *C.* x *sinensis* [13, 14]. Pathotypes represent phylogenetically-coherent lineages based on whole-genome sequencing (WGS) data or genotyping data [15–18]. These techniques have made it possible to identify previously unreported sublineages within pathotypes A and A*, which are responsible for outbreaks both in the pathogen’s area of origin or in regions where it has recently emerged [11, 16, 18].

Long-distance dissemination of *X. citri* pv. citri occurs primarily when humans transport diseased citrus plant material [19]. Since the early 2000s, there have been reports of several cases of geographical expansion and successful pathogen establishment in some Western and Eastern African countries, and the Caribbean [10, 20, 21]. *X. citri* pv. citri is a major threat to disease-free areas (e.g. New Zealand, Australia, and countries in Southern Africa and the Mediterranean) where it is listed as a quarantine organism. Preventing the establishment of *X. citri* pv. citri in new areas very much depends on the availability of specific detection protocols and the implementation of surveillance and quarantine measures. Given the significance of ACC, numerous molecular detection methods have been developed, (i) conventional PCR assays [22–27], (ii) real-time quantitative qPCR assays [28, 29]; and (iii) Lamp assay [30]. Issues of inclusivity (i.e., the ability of the assay to detect all strains of the target organism) and/or exclusivity (i.e., the capacity to generate negative responses from non-target strains) occur with most of the previous assays developed for *X. citri* pv. citri [31]. Therefore, we evaluated recently published diagnostic tools and developed a new system.

Many microbial genomes that are publicly available constitute a valuable resource for identifying new, specific molecular markers. In this study, we describe the development of highly specific conventional and real-time PCR assays from a DNA marker selected using in silico comparative genomic analysis of *X. citri* pv. citri genomes and non-target *Xanthomonas* genomes. The real-time PCR assay amplified an endogenous plant DNA sequence present in the sample (5.8S rDNA) as a co-extracted and co-amplified internal control. It reveals any flaws in DNA extraction and the presence of PCR inhibitors [32–34]. These protocols were further validated using naturally infected citrus material collected in the field. These new molecular tools were independently evaluated and compared to existing protocols by the French Agency for Food, Environmental and Occupational Health & Safety (ANSES).

## Results

### Selection of a specific DNA fragment for PCR and qPCR assay design

The comparative genomic analysis of 30 *X. citri* pv. citri genomes against 30 other *Xanthomonas* genomes using the MicroScope platform [35] resulted in the selection of 33 coding DNA sequences (CDS), which were present in all the *X. citri* pv. citri genomes and absent in the non-target genomes used (Table S1). CDS with sizes < 100 bp (*n* = 2) and CDS corresponding to mobile elements (integrases, transposases, phage- and plasmid-borne genes) were not considered further (*n* = 5). The CDS XAC1051 (564 pb) encoding for a putative transmembrane protein displayed the best in silico specificity among the remaining candidates. A homologous sequence was only found in the *X. citri* pv. cajani strain LMG 558. It was split into two fragments located on two different contigs of its draft sequence. Thus, XAC1051 was used to design the qPCR Taqman® assay and conventional PCR assays (Table 1). The Primer Express® software used to design systems could not generate an efficient probe/primer system to allow the lack of amplification of *X. citri* pv. cajani strains. Conversely, primers were successfully designed to prevent the target amplification in *X. citri* pv. cajani for the conventional PCR assay.

**Table 1:** Primers and probes used in this study

Primers/probes	Sequence 5′ > 3’	Amplicon size
**XAC1051-2qPCR**		
P-XAC1051-MGB (6-Fam™)	CGGTGAGAAGCTGTAC	58 bp
qPCR-XAC1051-F	AGAGGCGCACTATGGCTTTC	
qPCR-XAC1051-R	CAACCCAGGACCTGCAAGAA	
P-citrus5.8S- MGB (Vic™)	ATCCCGTGAACCATCG	94 bp
citrus5.8S -F	GCGAAATGCGATACTTGGTGTGA	
citrus5.8S-R	CGTGCCCTCGGCCTAATG	
**XAC1051-F/R PCR**		
XAC1051-F	AAATTCTTGTCGATCTGCTGGCT	499 bp
XAC1051-R	GCCGCCGCATAATTCTTCTCAC	

### Analytical specificity of XAC1051-2qPCR

The 58 bp targeted DNA region of *X. citri* pv. citri strain IAPAR 306, including the primers and probes perfectly matched (100% identity and 100% query coverage) with sequences belonging to all the 91 *X. citri* pv. citri genomes available on NCBI, three *X. citri* pv. citri historical genomes from herbarium samples used in this study and the *X. citri* pv. cajani strain LMG 558. Conversely, no significant similarity was found with sequences from the other 2790 non-target xanthomonads.

All *X. citri* pv. citri strains showed a FAM-positive signal when tested with the real-time PCR assay. The typical exponential amplification curves and Ct values ranged from 27.7 to 31.8 (mean of 29.7 and standard deviation of 1.0). Among the 101 non-target strains, only three strains of *X. citri* pv. cajani tested positive with the real-time PCR assay with Ct values ranging from 22.9 to 23.7.

### Dynamic range

**Fig. 1:**
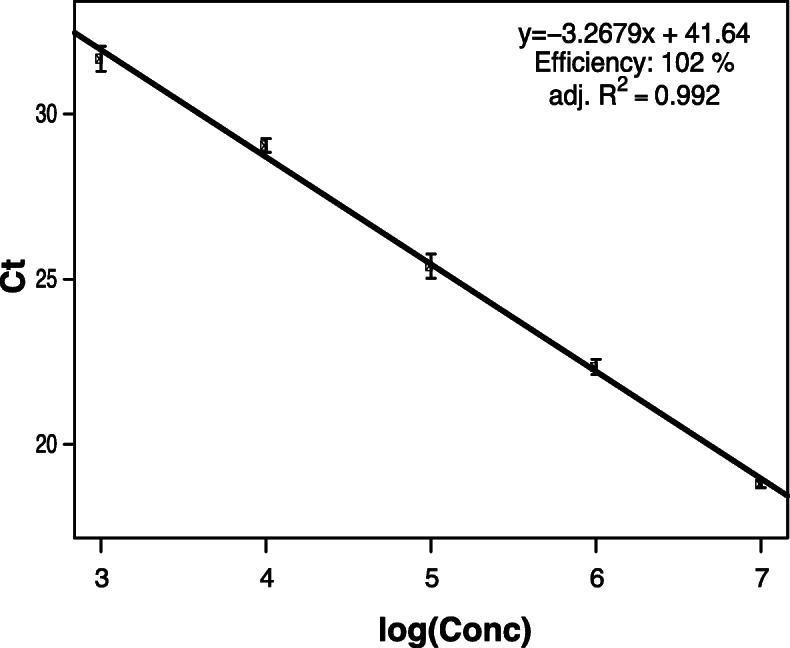
Standard curve obtained for a dilution series of the *X. citri* pv. citri strain IAPAR 306 in a sweet orange matrix. XAC1051-2qPCR was run on total DNA extracted from sweet orange leaves spiked with serially suspensions obtained from 10-fold serial dilution (10^7^ to 10^2^ CFU ml^− 1^). The standard curve was constructed using linear regression analysis of the threshold cycle (Ct) values for the serial dilutions over the Log_10_ of the initial target concentrations (compilation of all series and runs, corresponding to 9 replicates at each concentration level). The linear regression equation, the efficiency value and the adjusted R^2^ are indicated

The dynamic range of the duplex quantitative real-time PCR was assayed with three independent 10-fold dilution series of strain IAPAR 306 in each of the five different plant matrices. Calibration curves were constructed for each plant matrix by plotting the obtained Ct values against the Log_10_ of the standard concentrations (Fig. 1 and Fig. S1). A very strong linear relationship was observed between Ct values and the logarithm of bacterial concentration down to 1 × 10^3^ CFU ml-1 for all plant matrices with 0.988 > r^2^ > 0.992. The PCR efficiency (E) ranged from 90 to 102% according to the different plant matrices. As target concentration decreased, some of the replicate samples appeared to be negative (undetermined results): 22 negative signals out of 45 and 39 out of 45 registered for the concentrations of 10^2^ CFU ml^− 1^ and 10^1^ CFU ml^− 1^, respectively. Nevertheless, when the calibration curves included data from the 10^2^ CFU ml^− 1^ concentration, the coefficient and efficiency values were still acceptable (0.976 > r^2^ > 0.991 and 92% < E < 105%). In other words, the Ct values obtained for low target concentrations could be valid and may be considered as positive signals in routine diagnosis.

### Plant internal control

A VIC-positive signal, with Ct values ranging from 22.6 to 30.9, was detected for all symptomless (*n* = 90) and spiked plant samples with bacterial concentrations ranging from 1 × 10^1^ to 1 × 10^4^ CFU ml^− 1^ (*n* = 180). For higher bacterial concentrations, the number of VIC-positive signals decreased proportionally to the increase in bacterial populations: 15/36, 3/36 and 0/36 positive signals were registered for the concentrations of 1 × 10^5^ CFU ml^− 1^, 1 × 10^6^ CFU ml^− 1^ and 1 × 10^7^ CFU ml^− 1^, respectively.

### Analytical sensitivity in plant extracts

**Fig. 2:**
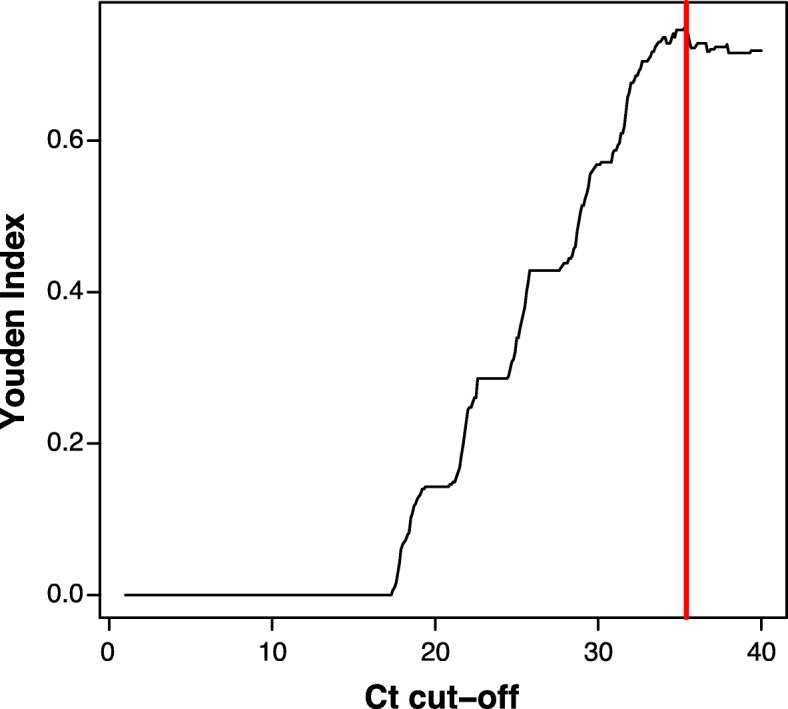
Determining the Ct cut-off according to the Youden index J. The optimal cut-off point is the PCR cycle with the highest value of the Youden index value, which represents a trade-off between sensitivity and specificity. The Ct cut-off was estimated to be 35.4

Before determining the analytical sensitivity, the first step was to implement a Ct cut-off, i.e., the value of Ct beyond which the real-time PCR signal was no longer considered as positive. Based on Youden’s index, the cut-off was estimated at 35.4 (Fig. 2). This cycle cut-off value was used to convert the quantitative data into binary data.

**Fig. 3:**
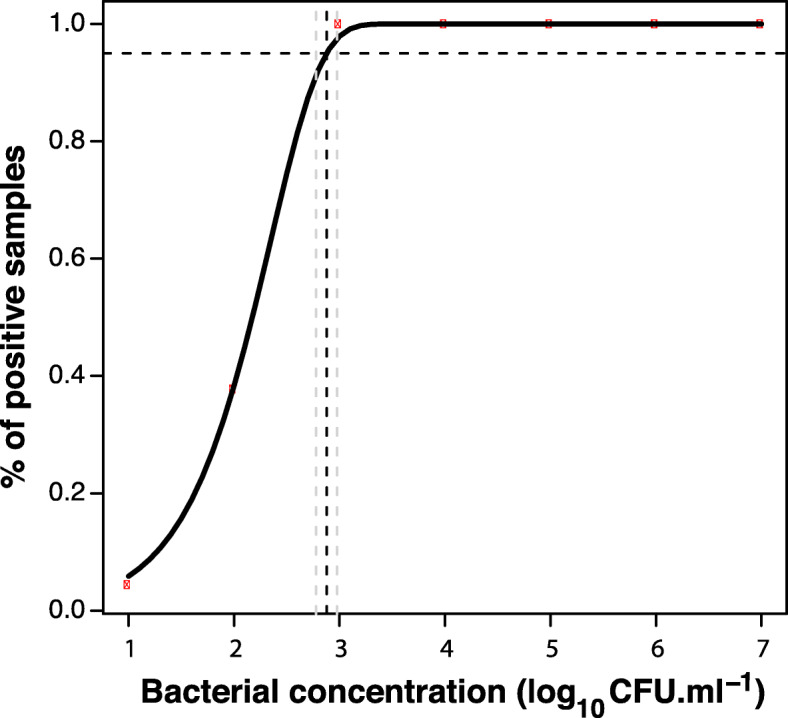
Determining the limit of detection (LOD95%) of the real-time qPCR assay. The x-axis represents the log of the bacterial concentrations, and the y-axis represents the probability of detection (POD) of replicate samples with a Ct value below 35.4 (cut-off). Each red point on the graph corresponds to means of 45 data samples. The smooth fitting line represents the best fitting model to the data points, based on a least squares approach using a probit model. The dark dotted vertical line indicates the bacterial concentration corresponding to LOD 95% (754 CFU ml^− 1^) and the two clear dotted lines indicate the corresponding 95% confidence interval

The limit of detection (LOD95%) was then determined for the whole dataset by plotting the numbers of positive responses obtained at the different concentrations against the Log_10_ of bacterial concentrations (Fig. 3). Log-Log was the best fitting curve with an estimated LOD95% of 754 CFU ml^− 1^ (95% CI 600–949), which corresponds to 15 bacteria per reaction. The same samples tested with the XAC1051-PCR assay yielded to an estimated LOD95% of 5234 CFU ml^− 1^ (95% CI 3656–7482), which corresponds to 105 bacteria per test sample.

### Repeatability, reproducibility and transferability

The intra-assay coefficients of variation obtained for all Ct value triplicates ranged from 0.035 to 2.482% with a median of 0.429%. The inter-assay coefficients of variations were calculated using the Ct values obtained for the three independent dilution series in each plant matrix and ranged from 0. 492 to 2.775% with a median of 1.558%. These low values reflected the repeatability and the reproducibility of the assay.

The qPCR efficiency values collected during the transferability assessment were all included between accepted range (90–110%) and all correlation coefficients were superior to 0.98 (Table 2). Low intra- and inter-assay *Cv* values were obtained for all experiments. Cut-off values were determined for each experiment involving a different qPCR device. These cut-off values were used to convert the quantitative data into qualitative data and to estimate the LOD95% values. LOD95% values were not statistically significant when the XAC1051-2qPCR protocol was tested on QS and LC480 devices.

**Table 2:** Characteristics of the XAC1051-2qPCR assay performed using three different devices

	Plant matrix	StepOnePlus	QS	LC480
**qPCR Efficiency**	**lemon**	93%	94%	97%
	**orange**	102%	103%	106%
**R**^**2**^	**lemon**	0.992	0.981	0.984
	**orange**	0.988	0.986	0.987
**Intra-assay variation Cv range (median)**		0.07–2.48% (0.34)	0.13–4.6% (0.60)	0.14–3.6% (0.81)
**Inter-assay variation Cv range (median)**		0.49–2.5% (1.50)	0.55–3% (1.30)	0.84–2.4% (1.50)
**Cut-off values**		35.9	38.43	38.89
**LOD95%** **(CI)**		2.90 (2.76–3.04)	3.04 (2.96–3.11)	3.09 (2.92–3.25)
**Vic Ct range**		25.8–31.2	22.3–27.9	23.4–29.7

### Detection from naturally infected fruit

In order to detect *X. citri* pv. citri from symptomatic fruits collected in the field, we used XAC1051-PCR, XAC1051-2qPCR and enumeration of *Xanthomonas*-like colonies on semi-selective agar medium (Table S2). All samples showing canker lesions were tested positive using these methods for all four assayed citrus lines, while all the healthy citrus control samples tested negative. Non-repeatable low qPCR signals (Ct > 35.4), interpreted as negative results, were obtained for some replicates of a few symptomless samples. Some doubtful *Xanthomonas* colonies were observed on KC medium for some tangor samples. Nevertheless, suspensions from these colonies were tested negative by XAC1051-2qPCR. The plant signal was detected for all symptomless samples, which confirmed that the failure to detect *X. citri* pv. citri was not due to a technical problem during the step of DNA extraction and/or PCR amplification.

### Detection from herbarium citrus samples

DNA extracted from the three herbarium specimens displayed substantial fragmentation (between 70 and 90 nt on average, see Table S3), as expected for DNA obtained from this type of material [36]. Nevertheless, they all tested positive with the Xac-qPCR assay. The presence of *X. citri* pv. citri in these samples was further confirmed by analyzing the next generation sequencing data (Table S3) obtained from the same samples during the course of another study (Rieux, unpublished data).

### Comparison of XAC1051-based conventional and real-time PCR assays with existing molecular tests

All *X. citri* pv. citri strains, irrespective of pathotype, tested positive with all five PCR assays and the three qPCR assays, with the exception of strain NCPPB 211 for J-Taqpth-qPCR (Table 3 and Table S4). The *X. citri* pv. aurantifolii B and C strains only tested positive only with the conventional Jpth1/2 and VM3/VM4 PCR assays and the VM-Syb-qPCR assay, which is consistent with previously published data [31].

**Table 3:** Comparison of the specificity of several PCR and real-time qPCR protocols

Assay		*X. citri* pv. citri			*X. citri* pv. aurantifolii (*n* = 5)	*X. euvesicatoria* pv. citrumelonis (*n* = 2)	Other *X. citri*^a^ pathovars (*n* = 9)	Other *Xanthomonas*^a^ species (*n* = 11)	Saprophytic xanthomonads^b^ (*n* = 15)
		A (*n* = 63)	A* (*n* = 11)	A^w^ (*n* = 4)					
PCR	Jpth1/2	62^c^	11	4	5	0	7	1	0
PCR	VM3/4	63	11	4	5	0	7	1	0
PCR	XACF/R	63	11	4	0	0	4	0	0
PCR	XCF/R	63	11	4	0	0	9	0	0
PCR	XAC1051-F/R	63	11	4	0	0	0	0	0
qPCR	XAC1051-2qPCR	63	11	4	0	0	1	0	0
qPCR	J-Taqpth-qPCR	63	11	4	0	0	3	0	0
qPCR	VM-Syb-qPCR	63	11	4	5	0	7	1	0

^a^ Isolated from *Citrus* spp. but not pathogenic to *Citrus* spp.

^b^ Isolated from *Citrus* spp.

^c^ Number of positive samples

In terms of exclusivity, the XAC1051-F/R PCR assay displayed 100% exclusivity whereas the other PCR assays picked up some non-target strains, with exclusivity values of 77.8% for both Jpth1/2 and VM 3/4 primers, 88.8% for XACF/R primers and 75.0% for XCF/R, respectively. Most of the PCR-positive non-target strains were phylogenetically close to *X. citri* [37]. Among the qPCR assays, XAC1051-2qPCR displayed the best exclusivity because only one non-target strain of *X. citri* pv. cajani was amplified, as seen above (97.2% exclusivity). J-Taqpth-qPCR showed an acceptable specificity (91.7% exclusivity) whereas VM-Syb-qPCR assay displayed only 77.8% exclusivity (i.e., the same value as for the conventional primer pairs).

The sensitivity of the different molecular assays was compared using different combinations of *X. citri* pv. citri strains and plant matrices (Table S5). Of the different conventional tests, the XAC1051-F/R assay was the most sensitive, with a detection threshold of 3 × 10^3^ ml^− 1^ in most of the plant matrices. A few samples were positive at 3 × 10^4^ ml^− 1^. The Miyoshi XCF/R protocol yielded similar results whereas all other conventional PCR assays were found less sensitive (3 × 10^4^ to 3 × 10^7^ CFU ml^− 1^, depending on plant matrices and strains tested). Cut-off thresholds were determined for each real-time protocol using the ROC method. As expected, real-time PCR assays displayed a higher level of sensitivity than the conventional PCRs with almost identical sensitivity (a majority of detection limits at 3 × 10^3^ CFU ml^− 1^).

## Discussion

*Xanthomonas citri* pv. citri is a major threat to global citrus production. The success of surveillance strategies and quarantine measures to control the international movement of *X. citri* pv. citri is highly dependent on the availability of rapid and reliable *in planta* detection tools. Previous studies have shown that a number of existing diagnostic protocols developed for *X. citri* pv. citri display insufficient exclusivity or in some cases inclusivity [31]. In this study, we developed new, highly specific and sensitive molecular assays to diagnose, detect and quantify *X. citri* pv. citri in citrus tissues. We compared the new assays to existing diagnostic tools using a broad collection of target and non-target strains and in different citrus matrices. Importantly, the present study evaluated how PCR protocols reacted to an extensive *X. citri* pv. citri strain collection, a feature that most earlier studies failed to achieve. Indeed, we assayed representative samples of all the lineages/sublineages of this bacterium, which have been reported to date throughout the world [11, 16–18, 38].

The selected target gene, XAC1051, encodes for a hypothetical transmembrane protein and is present on the chromosome of strains for which a complete genome sequence is available. This gene is part of a genomic region previously considered to be specific to *X. citri* pv. citri, when compared to *X. citri* pv *aurantifolii* B and C strains [39].

When a conventional PCR format was used, our assay appeared perfectly specific with 100% inclusivity and exclusivity, values that outcompete other conventional PCR assays.

Real-time quantitative PCR assays have significant benefits compared to conventional PCR, including shorter turnaround time, reproducibility and sensitivity. In addition, this method can be used for both qualitative and quantitative assessments (for recent examples, see [40, 41]). We therefore developed a duplex real-time PCR assay, targeting the same bacterial gene, and including a plant internal control. This control targets a plant 5.8S rDNA sequence conserved among *Citrus* species. We demonstrated successful amplification of the plant internal control for *X. citri* pv. citri concentrations ≤1 × 10^4^ CFU ml^− 1^. The plant signal was inhibited when higher bacterial concentrations were present in the extracts, which is consistent with previously published data [42]. Importantly, the plant control always yielded positive reactions in the absence of a bacterial signal. Therefore, it shows that a negative response for the bacterium is not due to a failure in the DNA extraction or PCR amplification process.

The XAC-1051-2qPCR assay was shown to be highly specific (100% inclusivity and 97.2% exclusivity). It displayed the best specificity when compared to the other real-time PCR assays available to date. Only strains of *X. citri* pv. cajani, responsible for bacterial leaf spot disease of pigeon pea (*Cajanus cajan*, Fabaceae) [43] tested positive with this molecular assay. This pathogen seems geographically restricted to India where pigeon pea is its sole known host species. *X. citri* pv. cajani was identified as the closest relative to *X. citri* pv. citri in a recent phylogenomic analysis [38], suggesting that this DNA region may have been present in their most recent common ancestor. If necessary, suspect samples can be confirmed by performing the XAC1051-F/R PCR assay.

When real-time quantitative PCR is used as a qualitative method, a Ct cut-off, i.e., the PCR cycle number above which any sample response value (Ct) is considered to be a false positive, must be set. Indeed, depending on experimental conditions, some false positives with high Ct values may be registered. These values result from a spontaneous increase in fluorescence background emissions and/or low-level DNA cross-contaminations [44, 45]. The cut-off varies depending on the experiment context: inherent characteristics of the real-time PCR system, qPCR instrument, qPCR mix and DNA template. A statistical approach based on ROC analysis, where both false positive and false negative qPCR signals are considered, allowed us to establish an optimal Ct cut-off. The estimated Ct cut-off was applied to determine the level of sensitivity of our assay in different plant matrices. The XAC-1051-2qPCR assay was shown to be highly sensitive with the capacity to detect approximatively 15 target cells per reaction. It demonstrated high repeatability and reproducibility. It also proved to be transferable between PCR cyclers with an optimization step, without compromising sensitivity and specificity. Importantly, the XAC-1051-2qPCR assay showed a good ability to detect the target from naturally infected citrus fruits. Interestingly, as predicted in silico, the XAC-1051-2qPCR assay was able to detect *X. citri* pv. citri from some herbarium samples dating back to 1911 (Table S3), despite the small quantities, the high fragmentation and the chemical modifications expected when ancient DNA (aDNA) is obtained from samples of this type [36]. The success of this assay is probably due to its high sensitivity and the small size of the target DNA (58pb). Interestingly, this result suggests that molecular tools, such as our XAC-1051-2qPCR assay, are useful for screening herbaria for plant pathogens. Screening is a prerequisite for further investigations such as metagenomics or population genetic analyses geared to reconstructing the evolutionary history of plant pathogens [46, 47].

## Conclusions

Herein, we conducted a thorough comparative analysis of several conventional and quantitative PCR protocols using the same strain collection and plant samples. Thus, we hope to provide end-users with precise information with regard to the respective advantages and limitations of the different protocols in order to help them select one or more complementary methods for testing plant material or microbial cultures.

In agreement with previous studies [48–50], we conclude that genome-informed identification of targets is a powerful aid when it comes to developing highly specific diagnostic techniques for plant pathogens.

## Methods

### Bacterial strains and culture conditions

Ninety-eight strains of *X. citri* pv. citri, representing the currently known genetic diversity of this pathovar were used in this study (Table S6). This collection included the strain LMG 696. This *X. citri* pv. citri pathotype A* strain was recently authenticated by WGS data, after initially being mislabeled as *X. campestris* pv. durantae [38].

The present study also examined 101 non-target strains representing other bacterial genera, other pathovars of X. *citri*, other *Xanthomonas* strains pathogenic to rutaceous species and saprophytic *Xanthomonas* strains isolated from citrus (Table S7).

To compare the different PCR and real-time quantitative PCR protocols (see 2.10 and 3.4), a specific collection of strains was used, including some of the strains of Tables S6 and S7. This collection is listed separately in Table S4 to facilitate comprehension.

Strains were stored at − 80 °C on beads in cryovials (Microbank Prolab Diagnostics) or freeze-dried for long-term storage. All strains were streaked on yeast-peptone-glucose agar (YPGA; yeast extract 7 g l^− 1^, peptone 7 g l^− 1^, glucose 7 g l^− 1^, and agar 18 g l^− 1^; pH 7.2) plates at 28 °C for 3–4 days to check for purity. Subcultures were produced from single colonies on YPGA plates incubated at 28 °C for 48 h. Bacterial suspensions were prepared and diluted in 0.01 M Tris buffer pH 7.2 (Sigma 7–9 Sigma-Aldrich, Saint-Quentin Fallavier, France) unless otherwise stated. Plant or canker lesion homogenates were prepared in the same buffer supplemented with 2% polyvinylpyrrolidone (PVP) with an average mol wt of 40,000 (Sigma-Aldrich).

### Selection of a DNA target specific to *Xanthomonas citri* pv. citri and a plant internal control

A preliminary bioinformatics screening of candidate CDSs was performed using the “gene phyloprofile” tool in the MicroScope platform (Genoscope, Evry, France) [35] on 30 *X. citri* pv. citri genomes, including pathotype A, A* and A^W^ strains against 30 non-target genomes of *Xanthomonas* (other species and pathovars). The aim was to select CDSs conserved in all *X. citri* pv. citri genomes that had limited or no identity to CDSs from non-target genomes present in the database. Then, using the selected nucleotide sequences as query, we performed BLASTn and Megablast searches against NCBI databases (February 2020): nr/nt, draft (*n* = 2225) and complete genomes (*n* = 565) of *Xanthomonas* group (taxid = 32,033), complete plasmids (*n* = 17,302) and complete bacteriophages (*n* = 2717). In silico presence and identity of chosen target regions was further confirmed in the three ancient genomes used in this study (see above, detection from herbarium citrus samples, and Table S3).

The 5.8S rDNA sequence from *C.* x *aurantiifolia* (MF797954) was selected to develop a endogenous plant internal control. This multicopy DNA region is conserved in the Rutaceae family, particularly among *Citrus* species*.*

### Duplex real-time quantitative PCR (XAC1051-2qPCR) and PCR assays

Taqman® probe and primers were designed from the *X. citri* pv. citri XAC1051 gene and the plant 5.8S rDNA sequence using Primer Express® software Version 3.0 (Applied Biosystems, Courtaboeuf, France) and were provided by Applied Biosystems (Courtaboeuf, France). The different Taqman® probe and primer systems were checked in Oligo 7.6 (Molecular Biology Insights, Inc., Cascade, CO, USA) in order to minimize interactions between the different oligonucleotides. The selected primers and probes are listed in Table 1.

Amplifications were carried out in 15-μl reaction volumes (in HPLC grade water) containing 7.5 μl of 2× Mastermix (Applied Biosystems), 600 nM of qPCR-XAC1051-F and qPCR -XAC1051-R primers, 425 nM of 5′FAM-labeled XAC-1051 MGB probe (P-XAC1051-MGB), 50 nM of citrus5.8SF and citrus5.8SR primers, 50 nM of 5′VIC-labeled citrus5.8S MGB probe (P-citrus5.8S- MGB) and 2 μl (pure bacterial suspensions) or 5 μl (total plant DNA extract) of template DNA.

The real-time PCR cycling conditions included a step at 50 °C for 2 min, an initial denaturation step at 95 °C for 2 min followed by 40 cycles of denaturation and annealing/elongation for 15 s at 95 °C and 1 min at 60 °C, respectively. Analyses were performed using the StepOnePlus software version v2.2.2. Each sample was at least duplicated.

Conventional primers were also designed from the XAC1051 gene using Oligo 7.6 (Table 1). Amplifications were carried out in 25-μl reaction volumes containing 5 μl of Green GoTaq® Reaction Buffer, 2 mM MgCl2, 0.5 μM of XAC1051-F and XAC1051-R, 0.2 mM each dNTP, 1.25 U of GoTaq® DNA Polymerase (Promega,) and 2 μl of template DNA. PCR amplifications were performed using a Veriti™ Thermal Cycler (Applied Biosystems, Courtaboeuf, France). The amplification program included denaturation at 95 °C for 2 min, 35 cycles consisting of denaturation at 95 °C for 45 s, annealing at 65 °C for 45 s, and extension at 72 °C for 1 min, and a final extension step at 72 °C for 5 min.

### Specificity of XAC1051-2qPCR assay

The in silico-determined specificity of the 58 bp target region from *X. citri* pv. citri was subject to an addition experimental check following the guidelines in the EPPO PM 7/98 (4) standard protocol [51]. The real time PCR protocol was assayed on pure cultures of target (*n* = 98) and non-target (*n* = 101) strains (Tables S6 and S7). Spectrophotometrically adjusted suspensions containing approx. 1 × 10^8^ CFU ml^− 1^ were diluted 100 or 10,000-fold for non-target and target strain assays, respectively. The suspensions were heated at 95 °C for 2 min and chilled on ice.

### Dynamic range in the plant matrix

The dynamic range of the real-time PCR assay, i.e., the range of initial template concentrations for which accurate Ct values are obtained, was determined on the dilution series of the strain IAPAR 306 in different citrus matrices: sweet orange (*C.* x *sinensis*), clementine mandarin (*C. reticulata*), grapefruit (*C.* x *paradisi*), lemon (*C.* x *limon*) and makrut lime (*C. hystrix*). Overnight cultures of IAPAR 306 were adjusted spectrophotometrically to a concentration of approx. 1 × 10^8^ CFU ml^− 1^ and serially 10-fold diluted. Fruit peel (0.1 g) was homogenized in 10 ml buffer using a grinder (Homex 6, Bioreba, Reinach, Switzerland) and spiked with bacterial suspensions at final concentrations ranging from 1 × 10^1^ to 1 × 10^7^ CFU ml^− 1^. Three replicated dilution series were performed in each citrus matrix. Total DNA was extracted from 2 ml homogenates using DNeasy Plant Mini kit (Qiagen, Courtaboeuf, France). Three qPCR replicates were carried out at each contamination level (nine Ct values were thus registered for each plant matrix and contamination level). Non-template controls (NTC) consisting of plant matrix and mix without DNA were included as negative samples (*n* = 18). Standard curves were generated for each citrus matrix by plotting Ct values against the logarithm of initial DNA concentrations. The reaction efficiency E was calculated according to the slope of the standard curves as follows: $E={10}^{\left(-\frac{1}{slope}\right)}-1$. The XAC1051-F/R PCR assay was also performed in duplicate using the same samples.

### Cut-off Ct value and limit of detection (LOD)

A ROC (Receiver operating characteristic) was used in order to determine the Ct cut-off value, i.e., the PCR cycle number above which signals are no longer interpreted as positive [52, 53]. This analysis, based on the determination of the Youden J index, which considers false positives and negatives [42, 54], was performed on Ct values obtained (see § 2.5. above) for samples with a priori positive status, i.e., citrus spiked samples with different bacterial concentrations (*n* = 135) and for samples with a priori negative status, i.e., NTC (*n* = 110). Samples with Ct values higher than the Ct cut-off value were then considered negative.

The analytical sensitivity of the XAC1051-2qPCR and the XAC1051-F/R PCR were estimated by determining the 95% limit of detection (LOD95%), i.e., the concentration at which a detection probability of 95% is expected [55–57] as explained previously [42].

### Repeatability, reproducibility and transferability

Repeatability (i.e., the level of agreement between replicates of a sample tested under the same conditions) and reproducibility (i.e., the ability of a test to provide consistent results when applied to aliquots of the same sample tested under different conditions (time, personnel, equipment, location, etc.)) were estimated according to the EPPO standard PM 7/76 (5) [58]. Repeatability was evaluated by computing intra-assay coefficients of variation ($Cv=\frac{\sigma }{\mu}\Big)$ based on Ct mean values of qPCR triplicates obtained for the concentrations ranging from 1 × 10^3^ CFU ml^− 1^ to 1 × 10^7^ CFU ml^− 1^ (70 *Cv* values). Reproducibility was evaluated on three qPCR runs independently performed for each plant matrix at different times. Inter-assay *Cv* values were calculated from the Ct values (PCR triplicate means) obtained for concentrations ranging from 1 × 10^3^ CFU ml^− 1^ to 1 × 10^7^ CFU ml^− 1^ (25 *Cv* values).

The protocol’s transferability was evaluated by reproducibility experiments performed by different operators, at different periods and in different laboratories (Cirad and ANSES). Dilution series of suspensions prepared from the IAPAR 306 strain in lemon and orange matrices already tested on the StepOnePlus device (Applied Biosystems) were also assayed using the Light Cycler LC 480 (Roche Life Science, Meylan, France) and the Quantstudio5 (QS5) (Applied Biosystems) real-time PCR systems. The application of the StepOnePlus master mix and real time cycling conditions for other devices gave poor results and required optimization (data not shown). Successful amplifications were obtained for both LC480 and QS5 devices when using the GoTaq® probe qPCR master mix kit (Promega) and the following cycling conditions: a step at 95 °C for 2 min followed by 45 cycles of denaturation and annealing/elongation for 15 s at 95 °C and 1 min at 60 °C, respectively. The concentrations of the different primers and probes remained the same as for the StepOnePlus. Efficiency and correlation coefficients were calculated for each fruit/real-time device data set. Cut-off values and LOD95% were estimated for each real-time qPCR device data set. Intra-assay and inter coefficients of variation were also calculated for each real-time qPCR device data set.

### Detection from naturally infected fruit

Fifteen fruit (several species) showing typical ACC symptoms were collected in citrus groves in Reunion (Table S2). Three lesions per fruit (0.1 g each) were sampled and independently homogenized in 10 ml buffer (45 samples). Fifty microliters were plated in duplicates on KC medium to estimate target concentrations [59]. Total DNA was extracted from 2 ml homogenates using DNeasy Plant Mini kit (Qiagen, Courtaboeuf, France). Fifteen citrus fruits showing no canker symptoms were analyzed as well, with two or three samples (same size as diseased samples) collected independently per fruit and processed as described above (43 samples). Conventional and real time quantitative PCR assays were performed on the different samples as described in § 2.3.

### Detection from herbarium citrus samples

The duplex PCR assay was also used to screen three herbarium citrus samples bearing typical citrus canker lesions, and collected between 1911 to 1992 in different areas (Table S3). They were provided by the Royal Mauritius Herbarium (acronym: MAU) and the National Herbarium of the Muséum National d’Histoire Naturelle, France (acronym: P). DNA extraction was performed in a bleach-cleaned facility room according to the protocol described in §2.5 using 0.01 g of leaf fragments (instead of fruit peel) as starting material. DNA concentration and fragment size were measured with Qubit (Invitrogen life Technologies) and TapeStation (Agilent Technologies) high sensitivity assays, respectively, according to the manufacturers’ instructions. The XAC1051-dqPCR assay was performed as described in § 2.3.

### Comparison of XAC1051-based conventional and real-time PCR assays with existing molecular tests

This comparison was performed by a laboratory (ANSES), which is different to the one where the qPCR XAC1051-based conventional and real-time PCR assays were developed (Cirad). We considered a selection of published PCR and real-time PCR protocols based on previously published data [31] or preliminary experimental and/or in silico data analyses (in the case of the most recent protocols). This excluded a recently published multiplex protocol designed to detect and differentiate between several citrus-associated xanthomonads [23], because the primers selected for *X. citri* pv. citri pathotype A perfectly matched in silico and reacted in preliminary assays with five other *Xanthomonas* citri pathovars. Table S8 presents the published protocols (and associated experimental conditions), which passed the first screen and were compared to the XAC1051-based conventional and real-time PCR assays in terms of analytical specificity and sensitivity.

In the first assay, bacterial suspensions in sterile distilled water (containing approx. 1 × 10^4^ or 1 × 10^6^ CFU ml^− 1^ for target and non-target strains, respectively) were used. A set of *X. citri* pv. citri strains (*n* = 78) and other xanthomonads (*n* = 42) (Table S4) was assayed in duplicate to compare the analytical specificity following the guidelines in EPPO PM 7/98 (4) standard protocol [51].

Then, the comparison of analytical sensitivity was carried out on different plant matrices spiked with tenfold-diluted bacterial suspensions. Plant matrices included the leaf or fruit peel of sweet orange grapefruit, lemon, Tahiti lime (*C.* x *latifolia*), clementine (*C.* x *clementina*) and makrut lime. They were spiked with 10-fold dilutions of the *X. citri* pv. citri strain CFBP 2525 with final concentrations ranging from 3 × 10^2^ to 3 × 10^7^ CFU ml^− 1^. In addition, leaves and fruit peels of Mexican lime (i.e., a host species susceptible to all *X. citri* pv. citri pathotypes) were spiked with 10-fold dilutions of the following strains: pathotype A strains JJ238–29 and LH001–1 (lineage 1 and 2, respectively), pathotype A^w^ strain LG115 (lineage 3), pathotype A* strain CFBP 2911 (lineage 4), the *X. citri* pv. aurantifolii pathotype B strain CFBP 2902 or the *X. citri* pv. aurantifolii pathotype C strain CFBP 2866 (same final concentrations as CFBP 2525). Homogenate production (0.1 mg plant matrix 5 ml buffer) and DNA extractions were performed as described above. PCR or real-time PCR assays were performed in duplicate.

## Supplementary information

**Additional file 1.**

**Additional file 2.**

## Data Availability

Available as Supplementary Material. R scripts available upon request.
