# Supplementary figures and images for: Development and comparative validation of genomic-driven PCR-based assays to detect Xanthomonas citri pv. citri in citrus plants
Source: BMC Microbiol. 2020 Oct 1;20:296. doi: 10.1186/s12866-020-01972-8 (PMC7528614; doi:10.1186/s12866-020-01972-8)

**clementine**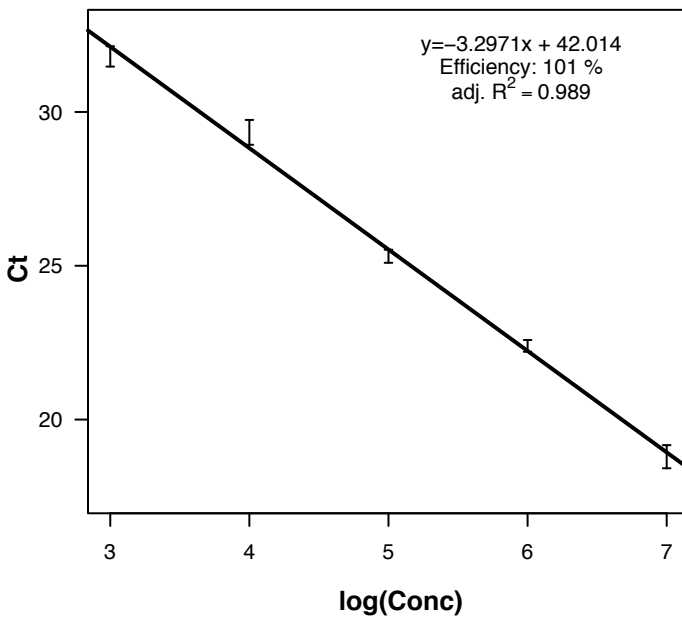**makrut lime**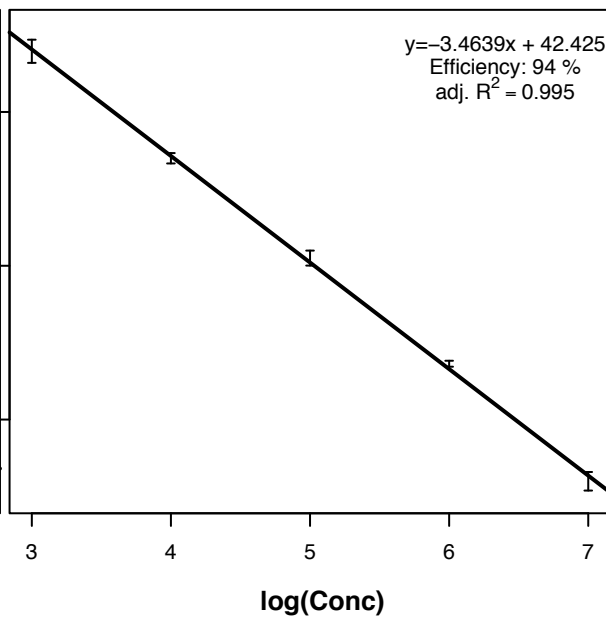**grapefruit**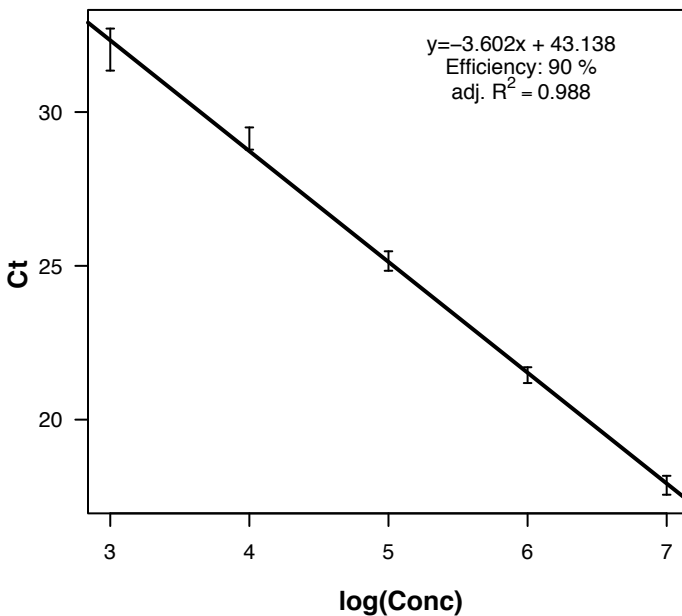**lemon**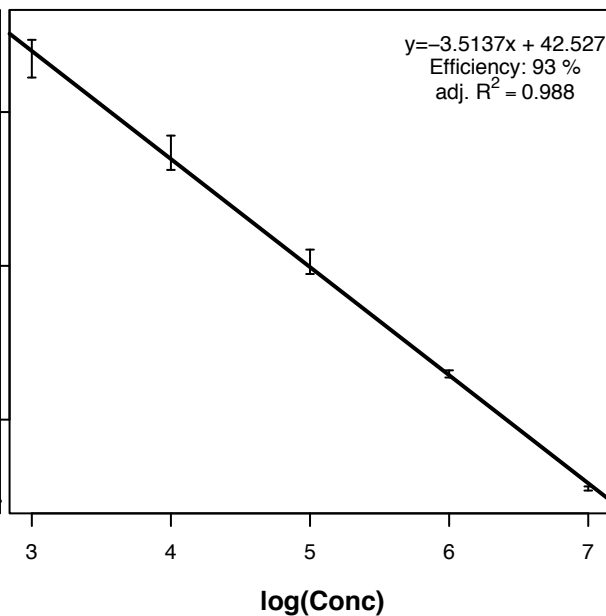

Supplement: Supplementary file 1 — Additional file 1. [file 12866_2020_1972_MOESM1_ESM.pdf]
