# Supplementary material for: Development and comparative validation of genomic-driven PCR-based assays to detect Xanthomonas citri pv. citri in citrus plants
Source: BMC Microbiol. 2020 Oct 1;20:296. doi: 10.1186/s12866-020-01972-8 (PMC7528614; doi:10.1186/s12866-020-01972-8)
Supplement: Supplementary file 2 — Additional file 2. [file 12866_2020_1972_MOESM2_ESM.pdf]

Table S1. ***X. citri*** **pv. citri genes (from strain IAPAR 306, chromosome NC_003919.1) evaluated by comparative genomics**

Gene number[a] (Start..End)	Hypothetical function	Size (bp)	Number of matching target genomes[b]	Matching non-target taxons[c]
XAC0147[d] (177709..179226)	RNA-directed DNA polymerase-retron type reverse transcriptase	1518	NC	NC
XAC1051 (1209505..1210068)	hypothetical protein	564	91 Xcc genome sequences, 99.82%-100% identity, 100% length	100% identity with *X. citri* pv. cajani on 100% sequence, but sequence divided in two fragments located on two different contigs
XAC1054 (1211424..1212428)	integrase	1005	NC	NC
XAC 1072 (1228276..1228677)	phage-related DNA-directed RNA polymerase	402	NC	NC
XAC1923 (2248775..2249779)	hypothetical protein	1005	91 Xcc genome sequences, 99.7%-100% identity, 100% length	99.7% identity on 100% sequence for *X. citri* pv. malvacearum, 99.8% identity on 100% sequence for *X. citri* pv. cajani
XAC1926 (2250926..2251294)	hypothetical protein	369	91 Xcc genome sequences, 99.7%-100% identity, 100% length	100% identity on 100% sequence for *X. citri* pv. malvacearum, *X. citri* pv. glycines, *X. citri* pv. cajani, *X. citri* pv. clitoriae, *X. citri* pv. leeana, *X. citri* pv. thespesiae, 99.7% identity on 100% sequence for *X. phaseoli* pv. phaseoli
XAC1927 (2251490..2252668)	Fe-S oxidoreductase	1179	91 Xcc genome sequences, 99.3%-100% identity, length from 74% to 100%	99.9% identity on 100% sequence for *X. citri* pv. glycines, *X. citri* pv. cajani, *X. citri* pv. clitoriae, and 99.8% for *X. citri* pv. leeana, *X. citri* pv. thespesiae, *X. phaseoli* pv. phaseoli, *X. citri* pv. punicae
XAC1928 (2252665..2253498)	hypothetical protein	834	91 Xcc genome sequences, from 99.9%-100% identity, length 100%	99%-99.4% identity on 100% sequence with *X. citri* pv. cajani, *X. citri* pv. glycines, *X. phaseoli* pv. phaseoli, *X. citri* pv. leeana, *X. citri pv.* thespesiae, *X. citri* pv. clitoriae, *X. citri* pv. punicae, *X. citri* pv. malvacearum

XAC2178 (2549428..2549655)	hypothetical protein	228	91 Xcc genome sequences, 100% identity, length from 98% to 100%	100% identity with *X. citri* pv. cajani, 94.7% on 100% sequence with X. *arboricola*, 94.3%-94.7% identity on 100% sequence with S*tenotrophomonas* spp., 95.1% identity on 100% sequence with *Alcaligenaceae* bacterium, and 94.3%-94.7% identity on 100% sequence with *Pseudomonas aeruginosa*
XAC2509 (2937638..2938138)	hypothetical protein	501	91 Xcc genome sequences, 100% identity, 100% length	100% identity on 100% sequence with *X. citri* pv. cajani, 97.6% identity on 100% sequence with *X. euvesicatoria* pv. citrumelonis
XAC2538 (2990993..2991265)	hypothetical protein	273	91 Xcc genome sequences, 99.8%-100% identity, 100% length	100% identity on 100% sequence with *X. citri* pv. glycines, 99.5%-99.6% identity on 100% sequence with *X. citri* [ex *Pseudomonas cissicola*], *X. citri* pv. vitiswoodrowii , *X. citri* pv. khayae, *X. phaseoli* pv. phaseoli, *X. citri* pv. punicae, *X. citri* pv. viticola, 98.6% identity on 100% sequence with *X. citri* pv. bilvae, 99% identity on 97% sequence with *X. citri* pv. aurantifolii
XAC4266 (5034781..5035413)	hypothetical protein	633	91 Xcc genome sequences, 99.8%-100% identity, 100% length	99.5% identity on 100% sequence with *X. citri* pv. cajani, *X. citri* pv. clitoriae, 99.4% identity on 100% sequence with *X. citri* pv. leeana, *X. citri* pv. thespesiae, *X. phaseoli* pv. phaseoli, *X. citri* pv. malvacearum, *X. citri* pv. khayae, *X. citri* pv. vitiscarnosae, *X. citri* pv. martyniicola
XAC4338 (5133650..5134021)	hypothetical protein	372	91 Xcc genome sequences, 100%-99.7% identity, 100% length	99.2% identity on 100% sequence with *X. citri* pv. mangiferaeindicae, *X. citri* pv. cajani, *X. axonopodis* pv. melhusii, *X. citri* pv. centellae, 98.9% identity on 100% sequence with *X. citri* pv. glycines
XANAC 0046 (46241..46828)	penicillin acylase	588	91 Xcc genome sequences, 100% identity, 100% length	99.8% identity on 100% sequence with *X. citri* pv. malvacearum, *X. citri* pv. leeana, *X. citri* pv. thespesiae, 99.6% identity on 100% sequence with *X. citri* pv. viticola, *X.axonopodis* pv. bauhiniae, *X. citri* pv. cajani, *X. citri* pv. vitistrifoliae, 99.5% identity on 100% sequence with *X. citri* pv. clitoriae, *X. citri* pv. vitiscarnosae, 99.3% identity on 100% sequence with *X. citri* [ex *Pseudomonas cissicola*] and *X.citri* pv. glycines

XANAC_0185 (185484..185705)	protein of unknown function	222	91 Xcc genome sequences, 100% identity, 100% length	100% identity on 100% sequence with *X. citri* pv. cajani, 99.5% identity on 100% sequence with *X. citri* pv. viticola, *X. citri* pv. khayae, *X. citri* pv. vitiscarnosae, *X. citri* pv. leeana, *X. citri* pv. martyniicola, *X. citri* pv. thespesiae, *X. euvesicatoria* pv. alfalfae
XANAC_0841 (850534..850740)	protein of unknown function	207	91 Xcc genome sequences, 100% identity, 100% length	100% identity on 100% sequence with *X. citri* pv. mangiferaeindicae, *X. citri* pv. melhusii and *X. citri* pv. centellae, 99.5% on 100% sequence with *X.citri* pv. glycines
XANAC_1218 (1213143..1213316)	protein of unknown function	174	91 Xcc genome sequences, 100% identity, 100% length	100% identity on 100% sequence with *X. citri* pv.cajani, 97.1% identity on 100% sequence with *X.citri* pv. glycines
XANAC_1219 (1213268..1213465)	protein of unknown function	198	91 Xcc genome sequences, 100% identity, 100% length	100% identity on 100% sequence with *X. citri* pv.cajani, 97.5% identity on 100% sequence with *X.citri* pv. glycines
XANAC_1231 (1219270..1219530)	exported protein of unknown function	261	91 Xcc genome sequences, 100% identity, 100% length	99.6% identity with *X. citri* pv. cajani on 100% length
XANAC_3035 (3073163..3073249)	DUF4189 domain-containing protein	87	NC	NC
XANAC_3193 (3204097..3204279)	protein of unknown function	183	91 Xcc genome sequences, 100% identity, 99%-100% length	99.4% identity on 100% length with *X. citri* pv. vitistrifoliae, 98.9% identity on 100% length with *X. citri* pv. glycines, *X. citri* pv. viticola, *X. citri* pv. cajani, *X. citri* pv. vitiscarnosae, *X. citri* pv. clitoriae, *X. citri* pv. martyniicola
XANAC_3524 (3545117.. 3545419)	protein of unknown function	303	91 Xcc genome sequences, 100% identity, 100% length	100% identity on 100% length with *X. citri* pv.cajani, 99.3% identity on 100% length with *X. citri* pv. clitoriae, 99% identity on 100% length with *X. citri* pv. bauhiniae, 98.7% identity on 100% length with *X. citri* pv. punicae, *X. citri* pv. glycines, *X. citri* pv. azadirachtae
XANAC_3628 (3676698..3676772)	protein of unknown function	75	NC	NC

XANAC_3637 (3682156..3682323	protein of unknown function	168	91 Xcc genome sequences, 100% identity, 100% length	100% identity on 100% length with *X. citri* pv.cajani, *X. citri* pv. glycines, *X. citri* pv. mangiferaeindicae, *X. citri* pv. bauhiniae, *X. citri* pv. vitiswoodrowii, *X. citri* pv. clitoriae, *X. citri* pv. centellae, *X. phaseoli* pv. phaseoli
XANAC_4546 (4613012..4613179)	protein of unknown function	168	91 Xcc genome sequences, 100% identity, 100% length	100% identity on 100% length with *X. citri* pv.cajani, 99.4% identity on 100% length with *X. citri* pv. clitoriae, 98.2% identity on 100% length with *X. citri* pv. vitiswoodrowii, 98.2% identity on 100% length with *X. citri* pv. khayae
XANAC_4715 (4764518..4764655)	protein of unknown function	138	91 Xcc genome sequences, 100% identity, 100% length	100% identity on 100% length with *X. citri* pv.cajani, 99.4% identity on 100% length with *X. citri* pv. clitoriae, 98.2% identity on 100% length with X. citri pv. vitiswoodrowii, 98.2% identity on 100% length with *X. citri* pv. khayae
XANAC_4813 (4882225..4882428)	protein of unknown function	204	91 Xcc genome sequences, 99.5%-100% identity, 100% length	100% identity on 100% length with *X. citri* pv. clitoriae, 99.5% identity on 100% length with X.citri pv. mangiferaeindicae, *X. citri* pv. bauhiniae, *X. citri* pv. khayae, *X. citri* pv. centellae
XANAC_5008 (5100670..5100870)	protein of unknown function	201	91 Xcc genome sequences, 99.5%-100% identity, 100% length	99.5% identity on 100% length with *X. citri* pv. glycines, *X. phaseoli* pv. phaseoli, 99% identity on 100% length with *X. citri* pv. melhusii, *X. citri* pv. punicae
XANAC_2436 (2458672..2458872)	protein of unknown function	201	91 Xcc genome sequences, 99.5%-100% identity, 100% length	100% identity on 100% length with *X. perforans*, *X.citri* pv. viticola, *X. citri pv. vitistrifoliae*, *X. phaseoli* pv. phaseoli , 99.5% identity on 100% length with X. *euvesicatoria*
XANAC_1716 (1723635..1724015)	protein of unknown function	381	91 Xcc genome sequences, 99.5%-100% identity, 100% length	100% identity on 100% length with *X. citri* pv. centellae, *X. citri* pv. mangiferaeindicae, 99.7% identity on 100% length with *X. citri* pv. vitistrifoliae, *X. citri* pv. melhusii
XAC2177 (2549611..2549979)	hypothetical protein	369	91 Xcc genome sequences, 100% identity, 90%-100% length	100% identity on 100% length with *X. citri* pv. cajani, 96.72% identity on 100% length with X. *arboricola*, 93.6%-92.8%identity on 100% length with *Stenotrophomonas maltophilia*

XACa0021 (11493..12713)	replication protein A	1221	NC	NC
XACb0016 (18214..19434)	replication protein A	1221	NC	NC

NC: not considered – see text for details.

[a] Name of predicted CDSs given by AMIGene (Annotation of Microbial Genes) tool of the MicroScope platform. The CDSs were classified into two categories, Common (XAC) or UNIQUE_AMIGA (XANAC), these last CDCs coding often for proteins of unknown function or having a low probability of coding for functional proteins.

[b] number of matching target genomes on 91 genome sequences of *X. citri* pv. citri available on NCBI (complete and draft genomes), including the genome of *X. campestris* pv. durantae reclassified as X. *citri* pv. citri : CP023662.1, CP023661.1, CP023285.1, CP020885.1, CP018858.1, CP018854.1, CP018850.1, CP018847.1, CP011827.2, CP008989.1, CP008995.1, CP009010.1, CP009013.1, CP009016.1, CP009022.1, CP008992.1, CP008998.1, CP009001.1, CP009004.1, CP009007.1, CP009019.1, CP009025.1, CP009028.1, CP006857.1, AE008923.1, CP020889.1, CP020882.1, CP009031.1, CP009034.1, CP009037.1, CP009040.1, CP003778.1, CP004399.1, MSQW01000001.1, MSQV01000001.1, LOKP01000122.1, LJGA01000001.1, LAUQ01000080.1, LAUP01000022.1, LAUN01000076.1, LAUG01000008.1, LAUF01000054.1, LAUE01000081.1, JRON01000085.1, JPYD01000355.1, CDMS01000294.1, CDMR01000279.1, CDMQ01000426.1, CDMO01000247.1, CDHD01000506.1, CDHC01000506.1, CDDV01000200.1, CDBA01000286.1, CDAX01000288.1, CDAV01000249.1, CDAT01000412.1,CDAS01000362.1 CDAQ01000377.1, CDAO01000405.1, CDAN01000306.1, CDAM01000677.1, CDAL01000823.1, CDAK01000289.1, CDAI01000120.1, CCXZ01000068.1, CCWY01000047.1, CCWX01000082.1, CCWK01000137.1, CCWJ01000067.1, CCWI01000065.1, CCWH01000089.1, CCWE01000066.1, CCWD01000068.1, CCWC01000056.1, CCWB01000074.1, CCWA01000113.1, CCVZ01000038.1, CCVY01000038.1, CCVX01000063.1, JPLP01000001.1, JPLO01000001.1, JPLN01000001.1, CDAY01000290.1, CDAW01000309.1, CDAU01000298.1, CDAP01000701.1, CDAJ01000134.1, CCWV01000180.1, CCWL01000073.1, CCWG01000142.1, CCWF01000115.1.

[c] These summarized the best hits obtained with BLASTn and Megablast searches against NCBI databases (nr, draft and complete genomes, complete plasmids, complete bacteriophages). *Xanthomonas* names according to Parkinson et al. (2009). Int. J. Syst. Evol. Microbiol. 59:264-274.

[d] This set of genes was obtained by testing 30 Xcc genomes against 30 non-target genomes using the MicroScope platform (Genoscope, France), and the tool “gene phyloprofile” in “comparative genomics”.

Table S2. Detection of *X. citri* pv. citri from field samples by XAC1051-2qPCR, XAC1051-PCR and isolation on KC semi-selective agar medium

Sample	citrus (cultivar)	qPCR results			Plating results	PCR results
Symptomatic fruits		Ct1	Ct2	conc. (CFU ml^{-1})	conc. (CFU ml^{-1})	
CG 1-1	*C.* x *aurantiifolia*	19.82	19.50	5.67×10^6	1.57×10^6	+
CG 1-2	*C.* x *aurantiifolia*	19.00	19.09	8.55×10^6	2.04×10^6	+
CG 1-3	*C.* x *aurantiifolia*	19.95	19.83	4.81×10^6	2.33×10^6	+
CG 2-1	*C.* x *aurantiifolia*	22.23	22.14	1.02×10^6	1.04×10^6	+
CG 2-2	*C.* x *aurantiifolia*	22.57	22.51	8.03×10^5	1.56×10^6	+
CG 2-3	*C.* x *aurantiifolia*	21.84	21.78	1.32×10^6	1.30×10^6	+
CG 3-1	*C.* x *aurantiifolia*	24.37	24.24	2.44×10^5	7.30×10^5	+
CG 3-2	*C.* x *aurantiifolia*	23.96	23.91	3.12×10^5	3.00×10^6	+
CG 3-3	*C.* x *aurantiifolia*	24.40	24.08	2.56×10^5	3.53×10^6	+
CG 4-1	*C.* x *aurantiifolia*	20.15	19.93	4.36×10^6	1.76×10^6	+
CG 4-2	*C.* x *aurantiifolia*	20.28	20.33	3.63×10^6	1.81×10^6	+
CG 4-3	*C.* x *aurantiifolia*	20.51	20.47	3.21×10^6	2.42×10^6	+
OTN 1-1	*C.* x *sinensis*	20.76	20.22	3.53×10^6	1.25×10^6	+
OTN 1-2	*C.* x *sinensis*	20.15	19.97	4.67×10^6	1.55×10^6	+
OTN 1-3	*C.* x *sinensis*	20.31	20.34	3.88×10^6	4.94×10^6	+
OTN 2-1	*C.* x *sinensis*	18.53	18.40	1.39×10^7	2.03×10^6	+
OTN 2-2	*C.* x *sinensis*	18.41	18.35	1.48×10^7	2.24×10^6	+
OTN 2-3	*C.* x *sinensis*	17.95	17.95	1.98×10^7	2.04×10^6	+

OTN 3-1	*C.* x *sinensis*	21.33	21.31	1.96×10^6	8.06×10^5	+
OTN 3-2	*C.* x *sinensis*	21.12	21.30	2.12×10^6	6.94×10^5	+
OTNT 3-3	*C.* x *sinensis*	20.64	20.49	3.30×10^6	6.78×10^5	+
OTN 4-1	*C.* x *sinensis*	16.21	15.86	7.42×10^7	3.86×10^6	+
OTN 4-2	*C.* x *sinensis*	16.46	16.31	5.81×10^7	3.60×10^6	+
OTN 4-3	*C.* x *sinensis*	15.90	15.82	8.29×10^7	1.81×10^6	+
MT 1-1	*C. reticulata*	19.04	19.02	9.45×10^6	9.90×10^6	+
MT 1-2	*C. reticulata*	18.86	18.65	1.15×10^7	7.72×10^5	+
MT 1-3	*C. reticulata* (Temple)	19.05	18.87	9.95×10^6	3.76×10^6	+
MT 2-1	*C. reticulata*	20.63	20.87	2.92×10^6	3.19×10^5	+
MT 2-2	*C. reticulata*	21.23	22.25	1.56×10^6	2.83×10^5	+
MT 2-3	*C. reticulata*	20.71	20.75	2.95×10^6	2.79×10^6	+
MT 3-1	*C. reticulata*	17.71	17.85	2.22×10^7	6.83×10^5	+
MT 3-2	*C. reticulata*	18.07	18.05	1.84×10^7	7.00×10^5	+
MT 3-3	*C. reticulata*	18.17	17.83	1.93×10^7	5.69×10^5	+
T 1-1	*C. reticulata* X *C.* x *sinensis*	17.87	17.86	2.10×10^7	2.36×10^6	+
T 1-2	*C. reticulata* X *C.* x *sinensis*	18.17	17.96	1.83×10^7	2.27×10^6	+
T 1-3	*C. reticulata* X *C.* x *sinensis*	17.96	17.9	2.13×10^7	2.14×10^6	+
T 2-1	*C. reticulata* X *C.* x *sinensis*	17.79	20.64	3.25×10^6	3.19×10^5	+
T 2-2	*C. reticulata* X *C.* x *sinensis*	20.49	20.29	3.72×10^6	3.06×10^5	+
T 2-3	*C. reticulata* X *C.* x *sinensis*	20.78	21.15	2.53×10^6	3.94×10^5	+
TA-1	*C. reticulata* X *C.* x *sinensis*	14.85	14.86	1.64×10^8	4.30×10^7	+

TA-2	*C. reticulata* X *C.* x *sinensis*	14.8	14.85	1.67×10^{8}	5.59×10^{7}	+
TA-3	*C. reticulata* X *C.* x *sinensis*	14.66	14.84	1.76×10^{8}	9.37×10^{7}	+
TA-4	*C. reticulata* X *C.* x *sinensis*	16.01	15.97	7.38×10^{7}	6.39×10^{7}	+
TA-5	*C. reticulata* X *C.* x *sinensis*	14.71	14.77	1.77×10^{8}	1.31×10^{8}	+
TA-6	*C. reticulata* X *C.* x *sinensis*	15.08	15.48	1.21×10^{8}	1.07×10^{8}	+

Table S3. Herbarium samples, XAC1051-qPCR and NGS results

Reference	Herbarium	Host	Origin	Year	DNA quantity (ng per mg of leaf)	DNA fragment length Mean [sd] (bp)	Xac1051-qPCR (Ct)	NGS*
P05297996	MNHN Paris	*Citrus* sp.	Philippines	1911	0,82	70 [19]	+ (31.1)	+ (1.6)
P05297992	MNHN Paris	*C.* x *limetta*	Philippines	1915	0,75	75 [22]	+ (33.6)	+ (8.5)
MAU0015160	Royal Mauritius Herbarium	*Citrus* sp.	Rodrigues	1992	1,12	90 [29]	+ (23.5)	+ (3.4)

* % of total reads mapping to *Xanthomonas citri* pv. *citri* reference genome (strain IAPAR 306, chromosome NC_003919.1).

Table S4. Analytical specificity of different conventional and real-time quantitative PCR assays determined from suspensions of *Xanthomonas* spp. pathogenic to citrus and non-target *Xanthomonas* strains

Strain	Taxon	Pathotype	Host	Origin	PCR					Real-time quantitative PCR		
					Jpth1/2	VM3/4	XAC F/R	XCF/R	XAC1051-F/R (this study)	XAC-1051-dqPCR (this study)	J-Taqpth-qPCR	VM-Syb-qPCR
**Target**												
CFBP 5234	*X. citri* pv. citri	A	*C.* x *limon*	Argentina	+	+	+	+	+	+(31.63)	+(26.58)	+(32.56)
JJ155	*X. citri* pv. citri	A	*C.* x *aurantiifolia*	Argentina	+	+	+	+	+	+(31.03)	+(28.76)	+(28.40)
LMG 9668	*X. citri* pv. citri	A	*C.* x *aurantiifolia*	Australia	+	+	+	+	+	+(31.24)	+(31.17)	ND
LMG 9672	*X. citri* pv. citri	A	*C.* x *aurantiifolia*	Australia	+	+	+	+	+	+(29.56)	+(28.38)	+(31.89)
LG097	*X. citri* pv. citri	A	*C.* x *limon*	Bangladesh	+	+	+	+	+	+(33.35)	+(31.41)	+(30.67)
LG101	*X. citri* pv. citri	A	*C.* x *aurantiifolia*	Bangladesh	+	+	+	+	+	+(28.99)	+(27.25)	+(29.07)
LG102	*X. citri* pv. citri	A	*Citrus* sp.	Bangladesh	+	+	+	+	+	+(30.05)	+(22.91)	+(25.60)
LG114	*X. citri* pv. citri	A	*C.* x *aurantiifolia*	Bangladesh	+	+	+	+	+	+(29.22)	+(27.87)	+(26.32)
LG117	*X. citri* pv. citri	A	*C.* x *aurantiifolia*	Bangladesh	+	+	+	+	+	+(33.29)	+(26.06)	+(28.87)
CFBP 2861	*X. citri* pv. citri	A	*C.* x *latifolia*	Brazil	+	+	+	+	+	+(28.89)	+(30.07)	+(26.68)
CFBP 2865	*X. citri* pv. citri	A	*C.* x *aurantiifolia*	Brazil	+	+	+	+	+	+(28.89)	+(31.03)	+(27.00)
CFBP 2908	*X. citri* pv. citri	A	*C. reticulata*	Brazil	+	+	+	+	+	+(31.59)	+(32.00)	+(29.50)
LG130	*X. citri* pv. citri	A	*Citrus* sp.	Brazil	+	+	+	+	+	+(33.16)	+(30.66)	+(29.66)
LJ303-01	*X. citri* pv. citri	A	*C. reticulata* X *C.* x *sinensis*	Burkina Faso	+	+	+	+	+	+(28.46)	+(26.34)	+(29.60)
LK004-2	*X. citri* pv. citri	A	*C.* x *paradisi*	Burkina Faso	+	+	+	+	+	+(30.20)	+(27.86)	+(31.49)
LK004-4	*X. citri* pv. citri	A	*C.* x *sinensis*	Burkina Faso	+	+	+	+	+	+(33.56)	+(30.60)	+(33.46)
LD071B	*X. citri* pv. citri	A	*Citrus* sp.	Cambodia	+	+	+	+	+	+(28.87)	+(32.84)	+(29.59)
JH081-2	*X. citri* pv. citri	A	*Citrus* sp.	China	+	+	+	+	+	+(32.95)	+(30.09)	+(30.5)
JJ035	*X. citri* pv. citri	A	*C. reticulata*	China	+	+	+	+	+	+(33.14)	+(31.10)	+(30.51)

CFBP 2857	*X. citri* pv. citri	A	*C.* x *aurantiifolia*	Fiji	+	+	+	+	+	+(32.67)	+(29.9)	+(30.21)
LL052	*X. citri* pv. citri	A	*C.* x *sinensis*	Grande Comore Is.	+	+	+	+	+	+(28.68)	+(29.27)	+(29.47)
JJ186-2	*X. citri* pv. citri	A	*Citrus* sp.	Guam	+	+	+	+	+	+(33.75)	+(30.97)	+(31.09)
NCPPB 1471	*X. citri* pv. citri	A	*C. maxima*	Hong Kong	+	+	+	+	+	+(28.98)	+(29.2)	+(29.51)
JJ165	*X. citri* pv. citri	A	*C. maxima*	India	+	+	+	+	+	+(30.61)	+(29.11)	+(30.68)
NCPPB 211	*X. citri* pv. citri	A	*Citrus* sp.	India	+	+	+	+	+	+(28.85)	-	+(30.74)
NCPPB 3610	*X. citri* pv. citri	A	*Poncirus trifoliata*	India	+	+	+	+	+	+(28.38)	+(25.92)	+(26.07)
LD108-1	*X. citri* pv. citri	A	*Citrus* sp.	Indonesia	+	+	+	+	+	+(32.12)	+(24.5)	+(28.90)
JJ163	*X. citri* pv. citri	A	*C. reticulata*	Japan	+	+	+	+	+	+(33.79)	+(29.05)	+(30.08)
JJ037-2	*X. citri* pv. citri	A	*C. medica* X *C. hystrix*	Malaysia	+	+	+	+	+	+(26.31)	+(30.29)	+(26.00)
JJ238-8	*X. citri* pv. citri	A	*C.* x *aurantiifolia*	Maldives Is.	+	+	+	+	+	+(31.93)	+(30.69)	+(32.44)
LE097-2	*X. citri* pv. citri	A	*C.* x *volkameriana*	Mali	+	+	+	+	+	+(29.62)	+(27.47)	+(25.30)
LE103-1	*X. citri* pv. citri	A	*C. reticulata* X *C.* x *paradisi*	Mali	+	+	+	+	+	+(29.78)	+(19.68)	+(25.90)
LE121-2	*X. citri* pv. citri	A	*C.* x *macrophylla*	Mali	+	+	+	+	+	+(32.13)	+(30.77)	+(29.46)
LH070-2	*X. citri* pv. citri	A	*C. aurantiifolia*	Mali	+	+	+	+	+	+(29.46)	+(28.14)	+(30.85)
LL077-7	*X. citri* pv. citri	A	*C.* x *latifolia*	Martinique Is.	+	+	+	+	+	+(30.10)	+(28.54)	+(29.76)
JK165	*X. citri* pv. citri	A	*C.* x *aurantium*	Mauritius Is.	+	+	+	+	+	+(32.19)	+(30.18)	+(29.94)
LJ228-1	*X. citri* pv. citri	A	*C. hystrix*	Mayotte Is.	+	+	+	+	+	+(28.04)	+(25.66)	+(28.92)
LD072-2	*X. citri* pv. citri	A	*Citrus* sp.	Myanmar	+	+	+	+	+	+(32.45)	+(29.84)	+(29.59)
CFBP 2525[PT]	*X. citri* pv. citri	A	*C.* x *aurantiifolia*	New Zealand	+	+	+	+	+	+(32.06)	+(30.29)	+(29.63)
JF090-12	*X. citri* pv. citri	A	*C.* x *aurantiifolia*	Oman	+	+	+	+	+	+(34.19)	+(31.51)	+(31.86)
JJ238-16	*X. citri* pv. citri	A	*C.* x *sinensis*	Pakistan	+	+	+	+	+	+(33.39)	+(27.99)	+(31.18)
LH001-1	*X. citri* pv. citri	A	*Citrus* sp.	Pakistan	+	+	+	+	+	+(32.96)	+(33.27)	+(30.38)
LE085-1	*X. citri* pv. citri	A	*C.* x *limon*	Papua New Guinea	+	+	+	+	+	+(33.57)	+(29.58)	+(30.97)
JK148-10	*X. citri* pv. citri	A	*C.* x *madurensis*	Philippines	+	+	+	+	+	+(29.63)	+(26.71)	+(26.35)

C21	*X. citri* pv. citri	A	*C.* x *sinensis*	Reunion Is.	+	+	+	+	+	+(30.54)	+(28.30)	+(29.19)
LH262	*X. citri* pv. citri	A	*C.reticulata* X *C.* x *sinensis*	Reunion Is.	+	+	+	+	+	+(30.45)	+(28.71)	+(32.40)
JJ010-7	*X. citri* pv. citri	A	*Citrus* sp.	Rodrigues Is.	+	+	+	+	+	+(31.12)	+(32.63)	+(32.09)
LH036-2	*X. citri* pv. citri	A	*C.* x *paradisi*	Senegal	+	+	+	+	+	+(29.04)	+(28.53)	+(30.40)
LJ002-2	*X. citri* pv. citri	A	*C.* x *sinensis*	Seychelles	+	+	+	+	+	+(31.42)	+(29.99)	+(34.79)
JK004-3	*X. citri* pv. citri	A	*C. reticulata x C.* x *sinensis*	South Korea	+	+	+	+	+	+(32.05)	+(30.06)	+(29.43)
LB234	*X. citri* pv. citri	A	*C. reticulata*	South Korea	+	+	+	+	+	+(28.48)	+(26.1)	+(31.26)
LB237	*X. citri* pv. citri	A	*C. reticulata*	South Korea	+	+	+	+	+	+(33.05)	+(30.21)	+(29.79)
JJ053-8	*X. citri* pv. citri	A	*C.* x *jambhiri*	Taiwan	+	+	+	+	+	+(29.67)	+(27.47)	+(26.38)
JK144-2	*X. citri* pv. citri	A	*C. hystrix*	Thailand	+	+	+	+	+	+(31.12)	+(31.47)	+(29.63)
JJ020-6	*X. citri* pv. citri	A	*Citrus* sp.	Thursday Is.	+	+	+	+	+	+(29.4)	+(29.94)	+(23.28)
NCPPB 3832	*X. citri* pv. citri	A	*C.* x *sinensis*	UK (interception)	+	+	+	+	+	+(29.09)	+(27.4)	+(27.72)
JJ162	*X. citri* pv. citri	A	*C.* x *paradisi*	Uruguay	+	+	+	+	+	+(32.70)	+(30.77)	+(29.56)
JJ238-29	*X. citri* pv. citri	A	*C.* x *sinensis*	Florida, USA	+	+	+	+	+	+(31.32)	+(31.58)	+(33.09)
JJ238-34	*X. citri* pv. citri	A	*C.* x *paradisi*	Florida, USA	+	+	+	+	+	+(31.81)	+(27.00)	+(27.56)
LMG 9322[T]	*X. citri* pv. citri	A	*C.* x *aurantiifolia*	Florida, USA	+	+	+	+	+	+(29.61)	+(30.43)	+(29.68)
LC002-1	*X. citri* pv. citri	A	*C. maxima*	Viet Nam	+	+	+	+	+	+(28.65)	+(27.05)	+(26.55)
JK004-9	*X. citri* pv. citri	A	*Citrus* sp.	Yemen	+	+	+	+	+	+(33.34)	+(29.46)	+(29.27)
LD071A	*X. citri* pv. citri	A*	*Citrus* sp.	Cambodia	+	+	+	+	+	+(27.70)	+(29.15)	+(32.00)
LL067-5	*X. citri* pv. citri	A*	*Citrus* sp.	Ethiopia	+	+	+	+	+	+(27.93)	+(29.60)	+(33.04)
CFBP 2858	*X. citri* pv. citri	A*	*C.* x *aurantiifolia*	Fiji	+	+	+	+	+	+(26.61)	+(30.73)	+(31.08)
NCPPB 3615	*X. citri* pv. citri	A*	*C.* x *aurantiifolia*	India	+	+	+	+	+	+(25.12)	+(26.51)	+(28.73)
JM047-2	*X. citri* pv. citri	A*	*C.* x *aurantiifolia*	Iran	+	+	+	+	+	+(28.03)	+(29.62)	+(32.97)
JS551	*X. citri* pv. citri	A*	*Citrus* sp.	Iran	+	+	+	+	+	+(26.70)	+(27.18)	+(31.28)
R-5228	*X. citri* pv. citri	A*	*Citrus* sp.	Iran	+	+	+	+	+	+(29.16)	+(21.59)	+(24.28)
CFBP 2911	*X. citri* pv. citri	A*	*Citrus* sp.	Pakistan	+	+	+	+	+	+(30.9)	+(32.72)	+(33.1)

JK002-17	*X. citri* pv. citri	A*	*C.* x *aurantiifolia*	Saudi Arabia	+	+	+	+	+	+(29.78)	+(31.15)	+(32.00)
JK143-10	*X. citri* pv. citri	A*	*C.* x *aurantiifolia*	Thailand	+	+	+	+	+	+(25.90)	+(27.72)	+(31.55)
NCPPB 3799	*X. citri* pv. citri	A*	*C.* x *aurantiifolia*	UK (interception)	+	+	+	+	+	+(27.82)	+(29.38)	+(32.01)
LG115	*X. citri* pv. citri	A^{w}	*C.* x *aurantiifolia*	India	+	+	+	+	+	+(28.90)	+(30.86)	+(31.24)
LG116	*X. citri* pv. citri	A^{w}	*C.* x *limon*	India	+	+	+	+	+	+(28.52)	+(29.74)	+(33.57)
JF090-8	*X. citri* pv. citri	A^{w}	*C.* x *aurantiifolia*	Oman	+	+	+	+	+	+(29.82)	+(32.03)	+(32.25)
LB305	*X. citri* pv. citri	A^{w}	*Citrus* sp.	Florida, USA	+	+	+	+	+	+(27.27)	+(29.07)	+(32.78)
LB302	*X. citri* pv. citri	A^{w}	*Citrus* sp.	Florida, USA	+	+	+	+	+	+(30.55)	+(29.13)	+(32.04)
**Other Xanthomonas species or pathovars pathogenic to citrus**												
CFBP 2904	*X. citri* pv. aurantifolii	B	*C.* x *limon*	Argentina	+	+	-	-	-	-	-	+(24.5)
JJ159	*X. citri* pv. aurantifolii	B	*C.* x *limon*	Argentina	+	+	-	-	-	-	-	+(27.71)
JJ161	*X. citri* pv. aurantifolii	B	*C.* x *limon*	Uruguay	+	+	-	-	-	-	-	+(28.33)
CFBP 2866	*X. citri* pv. aurantifolii	C	*C.* x *aurantiifolia*	Brazil	+	+	-	-	-	-	-	+(31.57)
CFBP 2905	*X. citri* pv. aurantifolii	C	*C.* x *aurantiifolia*	Brazil	+	+	-	-	-	-	-	+(29.96)
NCPPB 1759	*X. citri* pv. bilvae		*Feronia elephantacum*	India	+	+	+	+	-	-	+(20.73)	+(17.67)
NCPPB 3213	*X. citri* pv. bilvae		*Aegle marmelos*	India	-	-	+	+	-	-	-	-
CFBP 3138^{PT}	*X. euvesicatoria* pv. citrumelonis		*Poncirus trifoliata* X *C.* x *paradisi*	Florida, USA	-	-	-	-	-	-	-	-
JJ 238-28	*X. euvesicatoria* pv. citrumelonis		*Poncirus trifoliata* X *C.* x *paradisi*	Florida, USA	-	-	-	-	-	-	-	-
**X. citri pathovars not pathogenic to citrus**												
LMG 548^{PT}	*X. citri* pv. bauhiniae		*Bauhinia racemosa*	India	+	+	-	+	-	-	-	+(22.93)
LMG 558^{PT}	*X. citri* pv. cajani		*Cajanus cajan*	India	+	+	+	+	-	+(23.46)	-	+(22.93)
LMG 9045^{PT}	*X. citri* pv. clitoriae		*Clitoria biflora*	India	-	-	+	+	-	-	-	-
LMG 712^{PT}	*X. citri* pv. glycines		*Glycine max*	Sudan	+	+	-	+	-	-	-	+(24.28)
LMG 760	*X. citri pv.* malvacearum		*Gossypium* sp.	India	+	+	-	+	-	-	23.07	+(18.11)
LMG 761^{PT}	X. citri pv. malvacearum		*Gossypium* sp.	Sudan	+	+	-	+	-	-	-	+(21.00)

LH408	*X. citri* pv. mangiferaeindicae	*Mangifera indica*	India	+	+	-	+	-		-	+(21.59)
**Other *Xanthomonas* species**											
CFBP 2528^{T}	*X. arboricola* pv. juglandis	*Juglans regia*	New Zealand	-	-	-	-	-	-	-	-
LMG 7303^{PT}	*X. axonopodis* pv. begoniae	*Begonia tuberhybrida*	New Zealand	-	-	-	-	-	-	-	-
LMG 844^{PT}	*X. axonopodis* *pv.* phyllanthi	*Phyllanthus niruri*	Sudan	+	+	-	-	-	-	22.58	+(21.48)
LMG 568^{T}	*X. campestris* pv. campestris	*Brassica oleracea*	UK	-	-	-	-	-	-	-	-
CFBP 6107	*X. euvesicatoria* pv. allii	*Allium fistulosum*	Japan	-	-	-	-	-	-	-	-
CFBP 6775	*X. fragariae*	*Fragaria vesca*	Italy	-	-	-	-	-	-	-	-
CFBP 2532^{T}	*X. oryzae* pv. oryzae	*Oryza sativa*	India	-	-	-	-	-	-	-	-
LMG 695^{PT}	*X. phaseoli* pv. dieffenbachiae	*Anthurium* sp.	Brésil	-	-	-	-	-	-	-	-
CFBP 4641^{T}	*X. sacchari*	*Saccharum officinarum*	Guadeloupe	-	-	-	-	-	-	-	-
LMG 892^{PT}	*X. translu*cens pv. undulosa	*Triticum turgidum*	Canada	-	-	-	-	-	-	-	-
LMG 911^{T}	*X. vesicatoria*	*Lycopersicon esculentum*	New Zealand	-	-	-	-	-	-	-	-
**Saprophytic xanthomonads isolated from citrus**											
R-4918	*X. hortorum*	*Citrus* sp.	Iran	-	-	-	-	-	-	-	-
R-5430	*X. hortorum*	*Citrus* sp.	Iran	-	-	-	-	-	-	-	-
LMG 8989	*Xanthomonas* sp.	*Citrus* sp.	Florida, USA	-	-	-	-	-	-	-	-
LMG 8993	*X. arboricola*	*Citrus* sp.	Florida, USA	-	-	-	-	-	-	-	-
LMG 8998	*X. arboricola*	*Citrus* sp.	Florida, USA	-	-	-	-	-	-	-	-
LMG 9002	*Xanthomonas* sp.	*Citrus* sp.	Florida, USA	-	-	-	-	-	-	-	-
JK130-2	*Xanthomonas* sp.	*Poncirus trifoliata* X *C.* x *paradisi*	Argentina	-	-	-	-	-	-	-	-
JK130-3	*X. arboricola*	*C.* x *sinensis*	Argentina	-	-	-	-	-	-	-	-
JK130-4	*X. arboricola*	*C.* x *paradisi*	Argentina	-	-	-	-	-	-	-	-
JK130-5	*Xanthomonas* sp.	*C.* x *limon*	Argentina	-	-	-	-	-	-	-	-

JK130-7	*Xanthomonas* sp.	*C.* x *sinensis*	Argentina	-	-	-	-	-	-	-	-
JK130-8	*Xanthomonas* sp.	*C.* x *paradisi*	Argentina	-	-	-	-	-	-	-	-
JK130-10	*Xanthomonas* sp.	*C.* x *limon*	Argentina	-	-	-	-	-	-	-	-
JK130-13	*Xanthomonas* sp.	*C.* x *limon*	Argentina	-	-	-	-	-	-	-	-
JK130-15	*Xanthomonas* sp.	*C.* x *limon*	Argentina	-	-	-	-	-	-	-	-

[PT] pathotype strain; [T] type strain.
The strains common to Tables S6 and S7 are highlighted in grey.

Table S5. Threshold of detection of PCR and real-time qPCR assays targeting CBC strain, in different citrus matrices.

**Strain** (pathotype) 3x10^{2}- 3x10^{7} CFU/ml	**Citrus**	**Matrix**	**Conventional PCR (template qty)**					**Real-time qPCR (template qty)**		
			**Jpth1/2** Cubero et Graham, 2002 (2.5µl)	**VM3-4** Mavrodieva et al. 2004 (2µl)	**XAC F/R** Park (2006) (5µl)	**XCF/R** Miyoshi et al.,1998 (5µl)	**XAC1051 F/R** This study (2µl)	**XAC1051-2qPCR** This study (2µl)	**J-Taqpth** Cubero et Graham., 2005 (5µl)	**VM3-4** Mavrodieva et al. 2004) (2µl)
CFBP2525	Sweet orange	fruit	3 x10^{4} [a]	3 x10^{4}	3 x10^{5}	3 x10^{3}	3 x10^{3}	3 x10^{3}	3 x10^{3}	3 x10^{3}
**(A)**		leaf	3 x10^{5}	3 x10^{4}	3 x10^{5}	3 x10^{3}	3 x10^{3}	3 x10^{3}	3 x10^{3}	3 x10^{3}
	Lemon	fruit	3 x10^{5}	3 x10^{4}	3 x10^{4}	3 x10^{3}	3 x10^{3}	3 x10^{3}	3 x10^{3}	3 x10^{3}
		leaf	3 x10^{5}	3 x10^{4}	3 x10^{5}	3 x10^{3}	3 x10^{3}	3 x10^{3}	3 x10^{3}	3 x10^{2}
	Tahiti	fruit	3 x10^{5}	3 x10^{5}	3 x10^{5}	3 x10^{4}	3 x10^{4}	3 x10^{3}	3 x10^{3}	3 x10^{3}
	Lime	leaf	3 x10^{5}	3 x10^{4}	3 x10^{5}	3 x10^{4}	3 x10^{3}	3 x10^{3}	3 x10^{3}	3 x10^{3}
	Grapefruit	fruit	-	3 x10^{4}	3 x10^{4}	3 x10^{3}	3 x10^{3}	3 x10^{3}	3 x10^{3}	3 x10^{3}
		leaf	-	3 x10^{4}	3 x10^{4}	3 x10^{3}	3 x10^{3}	3 x10^{3}	3 x10^{3}	3 x10^{3}
	Clementine	fruit	3 x10^{5}	3 x10^{5}	3x10^{5}	3x10^{4}	3 x10^{3}	3 x10^{2}	3 x10^{3}	3 x10^{2}
		leaf	3 x10^{5}	3x10^{4}	3 x10^{4}	3x10^{3}	3 x10^{3}	3 x10^{3}	3 x10^{3}	3 x10^{3}
	Makrut	fruit	3 x10^{5}	3 x10^{4}	3 x10^{4}	3x10^{3}	3 x10^{3}	3 x10^{3}	3 x10^{3}	3 x10^{3}
	lime	leaf	3x10^{5}	3 x10^{4}	3 x10^{4}	3x10^{3}	3 x10^{3}	3 x10^{3}	3 x10^{3}	3 x10^{3}
LH1-1 **(A)**	Mexican	fruit	3 x10^{6}	3 x10^{6}	3 x10^{6}	3x10^{4}	3 x10^{4}	3 x10^{3}	3 x10^{3}	3 x10^{4}
	lime	leaf	3 x10^{7}	3 x10^{5}	3 x10^{4}	3 x10^{3}	3 x10^{3}	3 x10^{3}	3 x10^{3}	3 x10^{3}
JJ238-29		fruit	3 x10^{6}	3 x10^{6}	3 x10^{6}	3 x10^{5}	3 x10^{4}	3 x10^{4}	3 x10^{3}	3 x10^{4}
**(A)**		leaf	3 x10^{6}	3 x10^{4}	3 x10^{6}	3 x10^{4}	3 x10^{4}	3 x10^{3}	3 x10^{3}	3 x10^{3}
CFBP2911		fruit	3 x10^{6}	3 x10^{6}	3 x10^{5}	3 x10^{3}	3 x10^{3}	3 x10^{2}	3 x10^{4}	3 x10^{5}
**(A*)**		leaf	3 x10^{7}	3 x10^{6}	3x10^{5}	3 x10^{4}	3 x10^{4}	3 x10^{3}	3 x10^{4}	3 x10^{4}
LG115		fruit	3 x10^{5}	3 x10^{4}	3x10^{4}	3 x10^{3}	3 x10^{3}	3 x10^{3}	3 x10^{3}	3 x10^{3}
**(A[w])**		leaf	3 x10^{5}	3 x10^{4}	3x10^{4}	3 x10^{3}	3 x10^{3}	3 x10^{3}	3 x10^{3}	3 x10^{3}
CFBP2902		fruit	3 x10^{7}	3 x10^{6}	-	-	-	-	-	3 x10^{4}
**(B)**		leaf	3 x10^{7}	3 x10^{6}	-	-	-	-	-	3 x10^{5}
CFBP2866		fruit	3 x10^{6}	3 x10^{4}	-	-	-	-	-	3 x10^{4}
**(C)**		leaf	3 x10^{6}	3 x10^{6}	-	-	-	-	-	3 x10^{4}
Cut-off [b] values								36.5	35.5	33.5

[a] Threshold of detection (in CFU ml^{-1}): correct amplification for both duplicates of PCR and mean Ct values below the cut-off value estimated by ROC analyze for each qPCR assay on LC480.
[b] Cut-off values determined for each qPCR assay on LC480 using the ROC method.

Table S6. Strains of *Xanthomonas citri* pv. citri used for the determination of the specificity of XAC1051-2qPCR protocol.

Strain	Pathotype	Host	Origin	qPCR reponse (Ct)
JW160-1	A	*C.* x *aurantiifolia*	Bangladesh	+(29.24)
LG097	A	*C.* x *limon*	Bangladesh	+(29.62)
LG099	A	*C.* x *aurantiifolia*	Bangladesh	+(31.58)
LG101	A	*C.* x *aurantiifolia*	Bangladesh	+(31.25)
LG102	A	*Citrus* sp.	Bangladesh	+(30.36)
LG103	A	*C.* x *aurantiifolia*	Bangladesh	+(31.14)
LG104	A	*C.* x *aurantiifolia*	Bangladesh	+(30.19)
LG105	A	*C.* x *aurantiifolia*	Bangladesh	+(30.30)
LG106	A	*C.* x *aurantiifolia*	Bangladesh	+(30.24)
LG107	A	*C.* x *aurantiifolia*	Bangladesh	+(29.31)
LG108	A	*C.* x *aurantiifolia*	Bangladesh	+(29.29)
LG109	A	*C.* x *aurantiifolia*	Bangladesh	+(31.10)
LG110	A	*C.* x *aurantiifolia*	Bangladesh	+(30.83)
LG111	A	*C.* x *aurantiifolia*	Bangladesh	+(29.91)
LG112	A	*C.* x *aurantiifolia*	Bangladesh	+(30.58)
LG113	A	*C.* x *aurantiifolia*	Bangladesh	+(31.73)
LG114	A	*C.* x *aurantiifolia*	Bangladesh	+(31.48)
LG117	A	*C.* x *aurantiifolia*	Bangladesh	+(31.16)
IAPAR 306	A	*C.* x *sinensis*	Brazil	+(28.43)
IAPAR 12853	A	*Citrus* sp.	Brazil	+(29.14)
IBSBF 256	A	*C. reticulata*	Brazil	+(28.29)
IBSBF 1350	A	*C.* x *aurantiifolia*	Brazil	+(28.98)
LA081-8	A	*C.* x *sinensis*	Brazil	+(28.76)
LG130	A	*C.* x *aurantiifolia*	Brazil	+(28.46)
LD071B	A	*Citrus* sp.	Cambodia	+ (28.71)
JK004-1	A	*Citrus* sp.	China	+(28.79)
CFBP 1209	A	*C. maxima*	Hong Kong	+(29.46)
CFBP 2852	A	*Citrus* sp.	India	+(29.05)
NCPPB 211	A	*Citrus* sp.	India	+(30.21)
NCPPB 3562	A	*C.* x *limon*	India	+(29.28)
NCPPB 3610	A	*Poncirus trifoliata*	India	+(28.73)
NCPPB 3612	A	*C.* x *aurantiifolia*	India	+(30.02)
LD107-2	A	*C. maxima*	Indonesia	+(28.65)
CFBP 2855	A	*Citrus* sp.	Japan	+(28.86)
CFBP 2900	A	*Citrus* sp.	Japon	+(28.56)
JK146-4	A	*P. trifoliata*	Malaysia	+(29.17)
JJ238-10	A	*C.* x *aurantiifolia*	Maldives Is.	+(28.42)

LC080-2	A	*C. reticulata* X *C.* x *sinensis*	Mali	+(28.73)
LD007-1	A	*C.* x *aurantiifolia*	Mali	+(28.94)
LD007-2	A	*C.* x *aurantiifolia*	Mali	+(28.82)
LE117-1	A	C. x aurantiifolia	Mali	+(28.77)
LE116-1	A	*C.* x *aurantiifolia*	Mali	+(28.54)
CFBP 2525[PT]	A	*C.* x *limon*	New Zealand	+(28.78)
JJ238-16	A	*C.* x *sinensis*	Pakistan	+(31.22)
LH001-3	A	*Citrus* sp.	Pakistan	+(29.22)
JJ238-13	A	*Citrus* sp.	Philippines	+(29.28)
JK148-10	A	*C.* x *madurensis*	Philippines	+(29.75)
C40	A	*C.* x *sinensis*	Reunion Is.	+(28.62)
JJ010-1	A	*C.* x *aurantiifolia*	Rodrigues	+(27.68)
LH037-2	A	*C.* x *paradisi*	Senegal	+(29.07)
LH044	A	*C.* x *aurantiifolia*	Senegal	+(28.96)
LH041-1	A	*C. maxima*	Senegal	+(28.58)
LB100-1	A	*C. sinensis* X *P. trifoliata*	Seychelles	+(29.27)
LB232	A	*C.* x *limon*	South Korea	+(28.82)
CFBP 2548	A	*Citrus* sp.	Taiwan	+(28.71)
JK144-4	A	*C. reticulata*	Thailand	+(28.97)
LMG 9322[T]	A	*C.* x *aurantiifolia*	Florida, USA	+(29.24)
JN551-1	A	*C.* x *aurantiifolia*	Vietnam	+(29.55)
JK004-9	A	*Citrus* sp.	Yemen	+(31.82)
LD071A	A*	*Citrus* sp.	Cambodia	+(30.21)
LE003-1	A*	*C.* x *aurantiifolia*	Ethiopia	+(30.87)
LE020-1	A*	*C.* x *aurantiifolia*	Ethiopia	+(31.05)
LE032-1	A*	*C.* x *aurantiifolia*	Ethiopia	+(30.17)
LE065-1	A*	*C.* x *aurantiifolia*	Ethiopia	+(30.39)
NCPPB 3607	A*	*C.* x *aurantiifolia*	India	+(29.52)
NCPPB 3608	A*	*C.* x *aurantiifolia*	India	+(29.59)
NCPPB 3615	A*	*C.* x *aurantiifolia*	India	+(30.38)
LMG 696	A*	*Duranta repens?*[†]	India	+(29.74)
JS552	A*	*Citrus* sp.	Iran	+(29.72)
JS555	A*	*Citrus* sp.	Iran	+(29.34)
JS581	A*	*Citrus* sp.	Iran	+(30.11)
JS582	A*	*Citrus* sp.	Iran	+(30.28)
JS584	A*	*Citrus* sp.	Iran	+(30.41)
LH580	A*	*C.* x *aurantiifolia*	Iran	+(28.70)
LH620	A*	*C.* x *aurantiifolia*	Iran	+(29.66)
LH624	A*	*C.* x *aurantiifolia*	Iran	+(29.06)
LH627-2	A*	*C.* x *aurantiifolia*	Iran	+(30.78)
LH595	A*	*C.* x *aurantiifolia*	Iran	+(31.62)
LH605-1	A*	*C.* x *aurantiifolia*	Iran	+(28.32)

LJ235	A*	*Citrus* sp.	Iran	+(31.90)
LJ249	A*	*C.* x *aurantiifolia*	Iran	+(31.29)
LJ251	A*	*Citrus* sp.	Iran	+(31.39)
JF090-2	A*	*C.* x *aurantiifolia*	Oman	+(31.74)
JF090-3	A*	*C.* x *aurantiifolia*	Oman	+(29.28)
CFBP 2911	A*	*Citrus* sp.	Pakistan	+(28.93)
JK002-10	A*	*C.* x *aurantiifolia*	Saudi Arabia	+(29.74)
JK002-14	A*	*C.* x *aurantiifolia*	Saudi Arabia	+(29.02)
JK048	A*	*C.* x *aurantiifolia*	Saudi Arabia	+(29.86)
JK051	A*	*C.* x *aurantiifolia*	Saudi Arabia	+(29.13)
JM035-2	A*	*C.* x *aurantiifolia*	Saudi Arabia	+(29.92)
JJ238-24	A*	*C.* x *aurantiifolia*	Thailand	+(29.40)
JK143-11	A*	*C. maxima*	Thailand	+(29.57)
JK143-5	A*	*C.* x *aurantiifolia*	Thailand	+(29.52)
LB302	A^w	*Citrus* sp.	Florida, USA	+(30.31)
LB305	A^w	*Citrus* sp.	Florida, USA	+(30.11)
LG115	A^w	*C.* x *aurantiifolia*	India	+(30.28)
LG116	A^w	*C.* x *limon*	India	+(29.73)
JF090-8	A^w	*C.* x *aurantiifolia*	Oman	+(29.08)

† Host species as originally registered; Patané *et al*. (BMC Genomics 20:700) provide evidence that this strain was misidentified; ^{PT} pathotype strain; ^{T} type strain.
The strains used for comparison of the different PCR and qPCR protocols (Table S4) are highlighted in grey).

Table S7. Non-target strains used to evaluate the specificity of the XAC1051-2qPCRprotocol.

Strain	Taxon	Host of isolation	Country of origin	qPCR response (Ct)
**Other *Xanthomonas* species or pathovars pathogenic to citrus**				
JJ159	*X. citri* pv. aurantifolii (B)	*C.* x *limon*	Argentina	-
CFBP 2902	*X. citri* pv. aurantifolii (B)	*C.* x *limon*	Argentina	-
CFBP 2903	*X. citri* pv. aurantifolii (B)	*C.* x *limon*	Argentina	-
CFBP 2904	*X. citri* pv. aurantifolii (B)	*C.* x *limon*	Argentina	-
JJ160	*X. citri* pv. aurantifolii (B)	*Citrus* sp.	Uruguay	-
JJ161	*X. citri* pv. aurantifolii (B)	*C.* x *limon*	Uruguay	-
CFBP 2866	*X.citri* pv. aurantifolii (C)	*C.* x *aurantifolia*	Brazil	-
CFBP 2905	*X.citri* pv. aurantifolii (C)	*C.* x *aurantifolia*	Brazil	-
CFBP 2906	*X.citri* pv. aurantifolii (C)	*C.* x *aurantifolia*	Brazil	-
NCPPB 1759	*X. citri* pv. bilvae	*Feroniae elephantacum*	India	-
CFBP 2910	*X. euvesicatoria* pv. citrumelonis	*Poncirus trifoliata* X *C.* x *paradisi*	USA	-
CFBP 3114	*X. euvesicatoria* pv. citrumelonis	*P. trifoliata* X *C.* x *paradisi*	Florida, USA	-
CFBP 3138^{PT}	*X. euvesicatoria* pv. citrumelonis	*P. trifoliata* X *C.* x *paradisi*	Florida, USA	-
JJ238-20	*X. euvesicatoria* pv. citrumelonis	*C.* x *paradisi*	Florida, USA	-
JJ238-26	*X. euvesicatoria* pv. citrumelonis	*Citrus* sp.	Florida, USA	-
JJ238-27	*X. euvesicatoria* pv. citrumelonis	*P. trifoliata* X *C.* x *paradisi*	Florida, USA	-
JJ238-28	*X. euvesicatoria* pv. citrumelonis	*P. trifoliata* X *C.* x *paradisi*	Florida, USA	-
JJ238-31	*X. euvesicatoria* pv. citrumelonis	*C.* x *paradisi*	Florida, USA	-
JJ238-32	*X. euvesicatoria* pv. citrumelonis	*P. trifoliata* X *C.* x *paradisi*	Florida, USA	-
NCPPB 3213	*X. citri* pv. bilvae	*Aegle marmelos*	India	-
***X. citri* pathovars not pathogenic to citrus**				
LA098	*X. citri* pv. anacardii	*Anacardium occidentale*	Brazil	-
LA100	*X. citri* pv. anacardii	*Anacardium occidentale*	Brazil	-
LMG 532^{PT}	*X. citri* pv. aracearum	*Xanthosoma sagittifolium*	French West Indies	-
LMG 548^{PT}	X. citri pv. bauhiniae	*Bauhinia racemosa*	India	-
ICMP 7170	*X. citri* pv. cajani (9.5)	*Cajanus cajan*	Fiji	+ (22.93)
ICMP 9000	*X. citri* pv. cajani (9.5)	*Cajanus cajan*	Fiji	+ (23.66)
LMG 558^{PT}	*X. citri pv.* cajani (9.5)	*Cajanus cajan*	India	+ (23.75)
ICMP 1625	*X. citri* pv. cajani (9.6)	*Cajanus cajan*	India	-
ICMP 5849	*X. citri* pv. cajani (9.6)	*Cajanus cajan*	Sudan	-
LMG 7387	*X. citri* pv. cajani (9.6)	*Cajanus cajan*	Sudan	-
LMG 9045^{PT}	*X. citri* pv. clitoriae	*Clitoria biflora*	India	-
LMG 9046	*X. citri* pv. desmodiilaxiflori	*Desmodium laxiflorum*	India	-
LMG 7511	*X. citri* pv. fuscans	*Phaseolus* sp.	USA	-
LMG 712^{PT}	*X. citri* pv. glycines	*Glycine max*	Sudan	-

LMG 7429	*X. citri* pv. malvacearum	*Gossypium* sp.	Uganda	-
LMG 7430	*X. citri* pv. malvacearum	*Gossypium* sp.	Thailand	-
LMG 760	*X. citri* pv. malvacearum	*Gossypium* sp.	India	-
LMG 761^{PT}	*X. citri* pv. malvacearum	*Gossypium* sp.	Sudan	-
JF030-1	X.citri pv. mangiferaeindicae	*Mangifera indica*	Reunion	-
JN570	*X.citri* pv. mangiferaeindicae	*Mangifera indica*	Japon	-
LMG 8021^{PT}	*X. citri* pv. rhynchosiae	*Rhynchosia memnonia*	Sudan	-
LMG 867^{PT}	*X. citri* pv. sesbaniae	*Sesbania sesban*	NA	-
LMG 936^{PT}	*X. citri* pv. vignaeradiatae	*Vigna radiata*	Sudan	-
LMG 828	*X. citri pv. vignicola*	*Vigna unguiculata*	Sudan	-
**Other *Xanthomonas* species**				
LMG 494^{T}	*X. albilineans*	*Saccharum officinarum*	Fiji	-
LMG 852^{PT}	*X. arboricola* pv. pruni	*Prunus salicina*	New Zealand	-
LMG 539	*X. axonopodis* pv. axonopodis	*Axonopus scoparius*	Colombia	-
LMG 7303^{PT}	*X. axonopodis* pv. begoniae	*Begonia* sp.	New Zealand	-
LMG 7189	*X. axonopodis* pv. begoniae	*Begonia* sp.	USA	-
LMG 675^{PT}	*X. axonopodis pv.* cassiae	*Cassia tora*	India	-
LMG 691^{PT}	*X. axonopodis* pv. cyamopsidis	*Cyamopsis tetragonolobus*	India	-
LMG 693^{PT}	*X. axonopodis* pv. desmodiigangetici	*Desmodium gangeticum*	India	-
LMG 811^{PT}	*X. axonopodis* pv. patelii	*Crotalaria juncea*	India	-
LMG 844^{PT}	*X. axonopodis* pv. phyllanthi	*Phyllanthus niruri*	Sudan	-
LMG 849^{PT}	*X. axonopodis* pv. poinsettiicola	*Euphorbia pulcherrima*	India	-
LMG 955^{PT}	*X. axonopodis* pv. tamarindi	*Tamarindus indica*	India	-
LMG 8267	*X. bromi*	*Bromus willdenowii*	New Zealand	-
LMG 947^{T}	*X. bromi*	*Bromus carinatus*	France	-
LMG 535^{PT}	*X. campestris* pv. armoraciae	*Iberis* sp.	Tanzania	-
LMG 568^{T}	*X. campestris* pv. campestris	*Brassica oleracea*	United Kingdom	-
LMG 5267	*X. cassavae*	*Manihot esculenta*	Rwanda	-
LMG 673^{T}	*X. cassavae*	*Manihot esculenta*	Malawi	-
LMG 495	*X. euvesicatoria* pv. alfalfae	*Medicago sativa*	India	-
LMG 497^{PT}	*X. euvesicatoria* pv. alfalfae	*Medicago sativa*	Sudan	-
CFBP 6107	*X. euvesicatoria* pv. allii	*Allium fistulosum*	Japan	-
CFBP 6366	*X. euvesicatoria* pv. allii	*Allium cepa*	Reunion	-
LMG 8673	*X. melonis*	*Cucumis melo*	Brazil	-
CFBP 2532^{T}	*X. oryzae* pv. oryzae	*Oryza sativa*	India	-
LMG 797^{PT}	*X. oryzae* pv. oryzicola	*Oryza sativa*	Malaysia	-
LMG 695^{PT}	*X. phaseoli* pv. dieffenbachiae	*Anthurium* sp.	Brazil	-
LMG 8014	*X. phaseoli* pv. phaseoli	*Phaseolus vulgaris*	Romania	-
LMG 7455^{PT}	*X. phaseoli* pv. phaseoli	*Phaseolus vulgaris*	USA	-
CFBP 2603	*X. phaseoli* pv. manihotis	*Manihot esculenta*	Colombia	-

LMG 847[T]	*X. pisi*	*Pisum sativum*	Japan	-
LMG 472	*X. sacchari*	*Saccharum officinarum*	Guadeloupe	-
LMG 8684[T]	*X. theicola*	*Camellia sinensis*	Japan	-
LMG 896	*X. vasicola* pv. vasculorum	*Saccharum officinarum*	Puerto Rico	-
**Saprophytic xanthomonads isolated from citrus**				
JK130-1	*Xanthomonas* sp.	*C.* x *limon*	Argentina	-
JK130-2	*Xanthomonas* sp.	*P. trifoliata* X *C.* x *paradisi*	Argentina	-
JK130-3	*X. arboricola*	*C.* x *sinensis*	Argentina	-
JK130-4	*X. arboricola*	*C.* x *paradisi*	Argentina	-
JK130-5	*Xanthomonas* sp.	*C.* x *limon*	Argentina	-
JK130-6	*Xanthomonas* sp.	*C.* x *limon*	Argentina	-
JK130-7	*Xanthomonas* sp.	*C.* x *sinensis*	Argentina	-
JK130-8	*Xanthomonas* sp.	*C.* x *paradisi*	Argentina	-
JK130-9	*Xanthomonas* sp.	*C.* x *limon*	Argentina	-
JK130-10	*Xanthomonas* sp.	*C.* x *limon*	Argentina	-
JK130-11	*Xanthomonas* sp.	*C.* x *limon*	Argentina	-
JK130-12	*Xanthomonas* sp.	*C.* x *limon*	Argentina	-
JK130-13	*Xanthomonas* sp.	*C.* x *limon*	Argentina	-
JK130-14	*Xanthomonas* sp.	*C.* x *sinensis*	Argentina	-
JK130-15	*Xanthomonas* sp.	*C.* x *limon*	Argentina	-
R-5430	*X. hortorum*	Citrus sp.	Iran	-
R-5431	*X. hortorum*	*Citrus* sp.	Iran	-
R-5227	*X. hortorum*	*Citrus* sp.	Iran	-
R-4917	*X. hortorum*	*Citrus* sp.	Iran	-
R-4918	*X. hortorum*	Citrus sp.	Iran	-
LMG 8989	*Xanthomonas* sp.	*Citrus* sp.	Florida, USA	-
LMG 8992	*Xanthomonas* sp.	*Citrus* sp.	Florida, USA	-
LMG 8993	*X. arboricola*	*Citrus* sp.	Florida, USA	-
LMG 8998	*X. arboricola*	*Citrus* sp.	Florida, USA	-

[PT] pathotype strain; [T] type strain.
The strains used for comparison of the different PCR and qPCR protocols (Table S4) are highlighted in grey.

Table S8. Published protocols (sometimes slightly modified for improvement) used for the comparison with the XAC 1051-based conventional and real-time PCR assays developed in the study.

	Conventional PCR				SyberGreen Real-time PCR	Taqman Real-time PCR
**Reference publication**	Cubero and Graham 2002	Mavrodieva *et al.* 2004	Park *et al.* 2006	Miyoshi *et al.* 1998	Mavrodieva *et al.* 2004	Cubero and Graham 2005
**Primers**	Jpth1/2	VM3/4	XACF/R	XCF/R	VM3/VM4	J-RTpth3/4
**Cycler**	Veriti™ (Applied Biosystems)	Veriti™ (Applied Biosystems)	Vériti™ (Applied Biosystems)	Veriti™ (Applied Biosystems)	Light Cycler LC480 (Roche Life Science)	Light Cycler LC480 (Roche Life Science)
**Probe**	-	-	-	-	-	J-Taqpth2
**Conv. PCR mixtures**						
Buffer	1X	1X	1X	1X		
MgCl2 (mM)	3	1	3	2		
dNTPs (mM)	0.2	0.2	0.2	0.2		
Primer Forward (µM)	0.1	0.2	1	0.2		
Primer Reverse (µM)	0.1	0.2	1	0.2		
GoTaq G2 Hot start Polymerase Promega	1U	1U	1U	1U		
Template (µl)	2.5	2	5	5		
Final volume (µl)	25	25	25	25		
**Real-time PCR mixtures**						
GoTaq qPCR master mix Promega					1X	
GoTaq probe qPCR master mix Promega					-	1X
Primer Forward (µM)					0.5	0.4
Primer Reverse (µM)					0.5	0.4
TaqMan probe						0.2
Template (µl)					2	5
Final volume (µl)					20	25
**Conv. PCR cycles**						
Initial denaturation	94°C-5 min.	95°C-5 min.	94°C-5 min.	95°C-2.5 min.		
Cycles number	40x	40x	30x	32x		
Denaturation	[93°C-30s;	[95°C-45s;	[94°C-15s;	[95°C-1 min;		
Annealing	58°C-30s;	58°C-45s;	58°C-30s;	65°C-1 min.;		
Extension	72°C-45s]	72°C-45s]	72°C-30s]	72°C-2 min.]		
Final extension	72°C-10 min.	72°C-10 min.	72°C-7 min.	72°C-10 min.		
**Real-time PCR cycles**						
					Same conditions as in the original publication	Same conditions as in the original publication
